# A method of two-dimensional correlation spectroscopy combined with residual neural network for comparison and differentiation of medicinal plants raw materials superior to traditional machine learning: a case study on *Eucommia ulmoides* leaves

**DOI:** 10.1186/s13007-022-00935-6

**Published:** 2022-08-13

**Authors:** Lian Li, Zhi Min Li, Yuan Zhong Wang

**Affiliations:** 1 Medicinal Plants Research Institute, Yunnan Academy of Agricultural Sciences, Kunming, 650200 People’s Republic of China; 2 College of Traditional Chinese Medicine, Yunnan University of Chinese Medicine, Kunming, 650500 People’s Republic of China

**Keywords:** *Eucommia ulmoides* leaf, Two-dimensional correlation spectroscopy, Residual neural network, Drying methods, Geographical traceability

## Abstract

**Background:**

*Eucommia ulmoides* leaf (EUL), as a medicine and food homology plant, is a high-quality industrial raw material with great development potential for a valuable economic crop. There are many factors affecting the quality of EULs, such as different drying methods and regions. Therefore, quality and safety have received worldwide attention, and there is a trend to identify medicinal plants with artificial intelligence technology. In this study, we attempted to evaluate the comparison and differentiation for different drying methods and geographical traceability of EULs. As a superior strategy, the two-dimensional correlation spectroscopy (2DCOS) was used to directly combined with residual neural network (ResNet) based on Fourier transform near-infrared spectroscopy.

**Results:**

(1) Each category samples from different regions could be clustered together better than different drying methods through exploratory analysis and hierarchical clustering analysis; (2) A total of 3204 2DCOS images were obtained, synchronous 2DCOS was more suitable for the identification and analysis of EULs compared with asynchronous 2DCOS and integrated 2DCOS; (3) The superior ResNet model about synchronous 2DCOS used to identify different drying method and regions of EULs than the partial least squares discriminant model that the accuracy of train set, test set, and external verification was 100%; (4) The Xinjiang samples was significant differences than others with correlation analysis of 19 climate data and different regions.

**Conclusions:**

This study verifies the superiority of the ResNet model to identify through this example, which provides a practical reference for related research on other medicinal plants or fungus.

**Supplementary Information:**

The online version contains supplementary material available at 10.1186/s13007-022-00935-6.

## Background

*Eucommia ulmoides* Oliver is a unique tree species in China and belonging to *Eucommia* (Eucommiaceae), which is named as and Tuchong in Japanese and Du-Zhong in Chinese [1]. At present, it is widely planted in 27 provinces across the country, with a total cultivated area of 358,000 hectares, which accounts for more than 95% of the total resources of *E. ulmoides* in the world. The traditional uses of *E. ulmoides* peel are used as medicine with a history of more than 2000 years. It mainly contains chemical composition, including flavonoids, terpenoids, steroids, lignans, iridoid terpenoids, etc., which possesses pharmacological activity, such as blood pressure-lowering, blood sugar-lowering, blood lipids-regulating, anti-inflammation, liver protection, anti-cancer, prevention of osteoporosis and others [2–4]. This medicinal plant has a wide range of applications in chemical lines, national defense materials, aerospace sectors, transportations, communications, water conservancy, electric power, medical treatment, and so on [5]. However, the tree is dead due to the bark is peeled off, which is easy to cause a shortage of resources. With the rapid development of *Eucommia*-related industries, the demand for *Eucommia ulmoides* leaves (EULs) is also increasing as an important part of *Eucommia* resources. The EULs are rich in chemical components and can be harvested every year with great potential for development. Recent studies have shown that the chemical components and medicinal activity of its leaves are similar to those of bark [6, 7]. With the development of healthy food and raw materials for medicinal herbs, the production scale of EULs tea is the largest, currently accounting for more than 60% of the domestic output of *Eucommia* functional food (http://www.leadingir.com/trend/view/1525.html).

However, the quality of *E. ulmoides* medicinal materials is affected by many complex factors, such as genotype, cultivation technologies, growth conditions (sunlight, terrain, and climate), harvest time, processing, and storage conditions (humidity, time, temperature, etc.), cultivation environments, etc. These variable factors may cause changes in the chemical composition of *E. ulmoide*, leading to significant differences in their quality. Geographical variation is the main factor leading to differences in the chemical composition of EULs. And most authentic traditional herbal medicines contain enough active chemical components. Herbs grown in different environments can produce various secondary metabolite components, leading to differences in their intrinsic qualities. In addition, since EULs are mostly picked when the branches and leaves are lush in summer (June, July, and August) and autumn (September, October, and November), the volume is fluffy. After being placed, fresh leaves easily change color and taste, so they need to be processed in time. Different drying methods have their advantages in terms of drying cost, time, convenience, efficiency, and environmental impact. Three methods were commonly applied to plant drying, including natural, artificial, and comprehensive methods. Traditional drying methods are conducted in the shade, air, or sun. The drying method of EULs is dried at low temperature or drying, while there is no specific temperature limit, which has been listed in “China Pharmacopoeia” [8]. The traditional methods of drying EULs are the shade and sun. The advantages of drying in the shade and sun are simple operation, low cost, and no need for a dryer. The weather does not restrict the drying methods at different temperatures (40 ℃ and 60 ℃), and the equipment is simple and can be used for large-scale production. Therefore, we compared different gradients of natural drying methods including shade drying, sun drying, 40 °C and 60 °C, providing a theoretical basis and others to choose methods for drying EULs. It is urgent to find a fast and effective identification method to evaluate different drying methods and regions of EULs. This is of great significance for ensuring the quality of traditional Chinese medicine (TCM) to ensure the efficacy of proprietary Chinese medicines and the healthy development of the Chinese medicine market. Therefore, the different drying methods and regions of EULs were compared and evaluated in this study, which provides a theoretical basis for finding a scientific drying method and the most suitable regions. It is necessary to use modern technology to achieve this.

Traditional methods of identification technology such as nuclear magnetic resonance (NMR), high-performance liquid chromatography (HPLC), and mass spectroscopy (GC-MS, LC-MS) are generally applied to distinguish in foods, medicine, etc. Although these techniques are sensitive and accurate, they require costly instrumentation and maintenance, trained personnel to operate the instruments, and are time-consuming. From an economic point of view, this makes them less attractive as a technique for determining identification. As an alternative technology, spectroscopic technologies have the characteristics of sample pretreatment is simple, routine analysis is fast, operation is simple and easy, no reagents are required, and economical technique, which allows simultaneous multi-component analysis. It has been widely used in the fields of textile, environment, petroleum, agriculture, medicine, and so on [9, 10]. Although some spectroscopic technology (Raman spectra, etc.) are applied to identify herbs or medicines, portable Fourier transform near infrared spectroscopy (FT-NIR) has benefits over the already investigated technology that was applied to the metabolic fingerprinting analysis, and a great advantage of which is rapid and non-destructive analysis. Spectroscopy technology is used in conjunction with chemometrics. Chemometrics is crucial for mining the most valuable data and building high-performance models. Traditional chemometrics contains support vector machines, partial least squares discriminant analysis (PLS-DA), etc. [11–13]. In addition, with the development of the era of big data, artificial intelligence has an irreplaceable role in data analysis and its visualization. Scholars is used artificial intelligence algorithms in the prediction of sugar content, which was only a pioneering example in the food field [14]. In recent years, deep learning technology have widely used in remote sensing research, including the field of the regions and species of speech recognition, TCM, and food [15]. The convolutional neural network (CNN) is a deep learning algorithm that is mainly used for image recognition and is widely used in the field of medical image analysis [16]. Residual Neural Network (ResNet), as an excellent CNN network, has good convergence and accuracy, which applies the stochastic gradient descent method to update the weights [17]. It can optimize gradient vanishing and exploding in ResNet, which has been successfully applied to TCM and fungus based on two dimensional correlation spectroscopy (2DCOS) images, including the species, storage period, geographical traceability, etc. [12, 18, 19]. 2DCOS can effectively solve the spectral overlap and improve the apparent spectral resolution. These spectral types include not only IR, but also expanded to Raman spectroscopy, ultraviolet spectroscopy, etc. Yue et al. proved that a superior method verification of deep learning combined with 2DCOS in the identification of different regions and parts of *Paris* polyphylla var. *yunnanensis* than the traditional model, such as support vector machines, PLS-DA [20]. A study showed that 20 *Dendrobium* species have successfully identified by two-dimensional correlation spectroscopy combined with ResNet based on feature bands extracted by spectrum standard deviation [21]. In previous studies, although the performance of ResNet models is superior to traditional models, complex operations such as spectral preprocessing and feature band extraction are required. In addition, there are few reports on EULs. This work directly uses two-dimensional spectral images for ResNet modeling and compares traditional models (PLS-DA) without preprocessing and feature extraction, which reduces the complexity of data processing.

In this study, the FT-NIR technology combined with ResNet and chemometrics to identify the different drying methods and geographical traceability of EULs, and the specific components of this study were: (1) to collect the FT-NIR spectral data of 534 EULs samples; (2) to explore the distribution trends of various samples with exploratory analysis of principal component analysis (PCA) and hierarchical cluster analysis (HCA); (3) to obtain three two-dimensional correlation spectroscopy (2DCOS) of Synchronous, asynchronous, and integrated images. (4) to establish and compare two models of PLS-DA and ResNet for identifying with different drying methods and regions of EULs samples; (5) Correlation analyses of 16 different region s and 19 climate data were performed and analyzed. Therefore, image processing of ResNet is extended to different research directions of medicinal plants or fungus, which may provide more concise methods without less loss of classification ability.

## Materials and methods

### Sample information

**Fig. 1:**
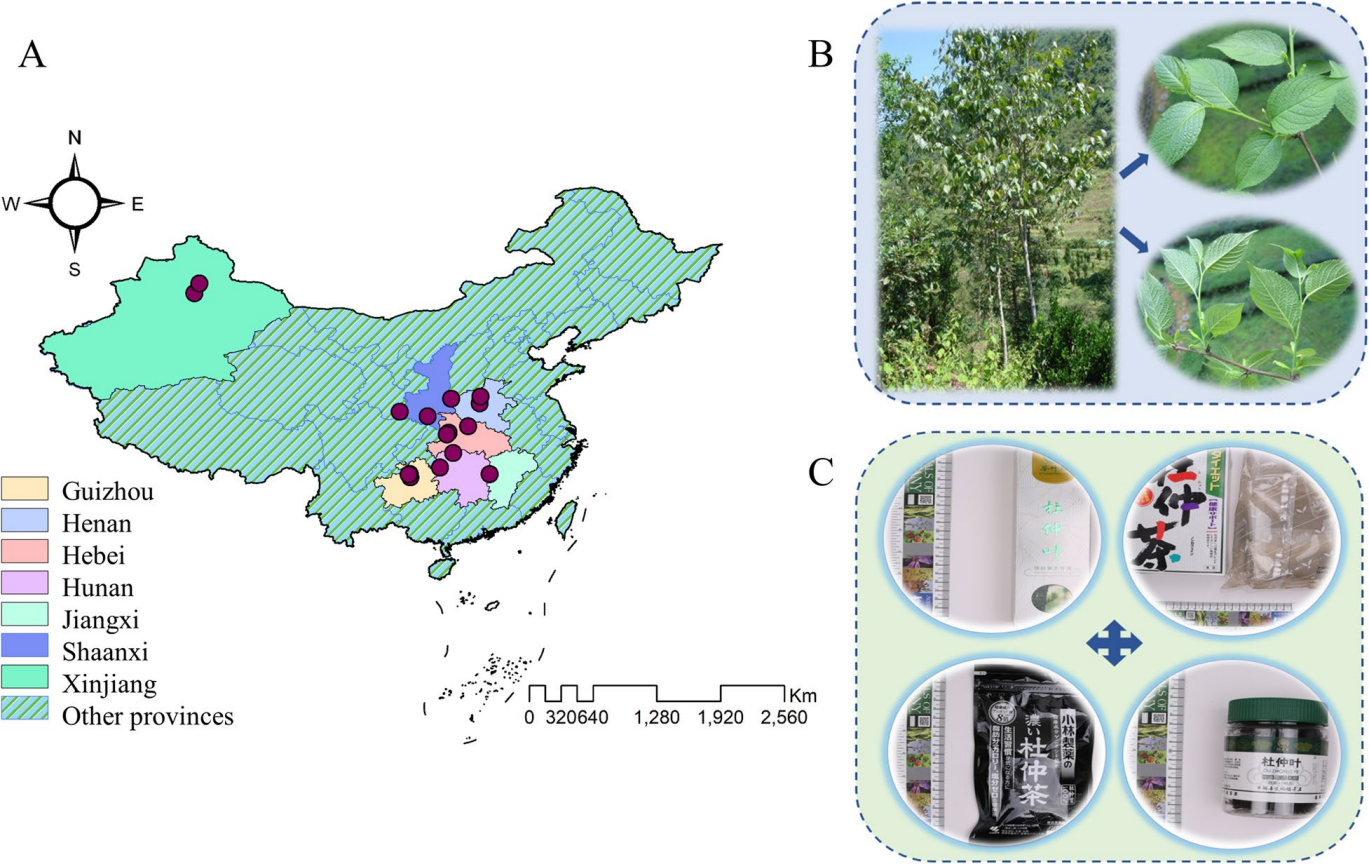
**A** Distribution of samples of *E. ulmoides* leaves in China; **B** The shape of *E. ulmoides* leaves; **C** The processed products of *Eucommia*

A total 534 samples of EULs were collected in the second quarter of summer from seven provinces in China. The details sample information was displayed in Fig. 1 and Table 1. All 534 samples were identified by Professor Ke-Gang Li (Jishou University, Hubei, China). To avoid the influence of individual differences in samples due to collection in different areas (upper, middle, and lower of the canopy), all samples were collected from the middle layer of the treetop, including both sunny and shady sides. All the samples were divided into four parts and processed according to different drying methods. Some EULs were dried in an oven at 40 °C (136 samples) and 60 °C (133 samples) to constant weight, while the remaining samples were dried in the shade drying (132 samples) and natural sun drying (133 samples). The dried EULs were passed through high-speed grinders and sieved with 80-mesh stainless steel (to avoid flocculent *E. ulmoides* gum interfering with component detection). The sieved powders were placed in ziplock bags and stored at room temperature in the dark for subsequent experiments.

**Table 1:** Information of the *E. ulmoides* leaves samples

Region	Collection site	Elevation (m)	Latitude (N)	Longitude (E)	Numbers	40℃	60℃	Shade drying	Sun drying	Total
Guizhou	Shangji Town, Bozhou District, Zunyi City, Guizhou Province	857	27°24′ 04.63′′	106°57′ 42.17′′	40	10	10	10	10	121
	Gaoqiao Town, Huichuan District, Zunyi City, Guizhou Province	946	27°43′ 29.82′′	106°52′ 49.39′′	40	10	10	10	10	
	Xiangkou Town, Honghuagang District, Zunyi City, Guizhou Province	925	27◦38′ 42.25′′	106°53′ 35.42′′	41	11	10	10	10	
Henan	Liujiahe Town, Xingyang City, Henan Province	252	34°04′ 21.02′′	113°12′ 52.28′′	39	10	10	9	10	119
	Jingya Forest Farm, Cili County, Zhangjiajie City, Hunan Province	420	34°43′ 10.21′′	113°17′ 18.14′′	40	10	10	10	10	
	Zhaiyang Township, Jishou City, Hunan Province	334	29°31′ 22.69′′	110°46′ 02.50′′	40	10	10	10	10	
Hubei	Muyu Town, Shennongjia Forestry District, Hubei Province	1343	31°28′ 44.43′′	110°22′ 43.08′′	12	3	3	3	3	64
	Xiangfan District, Xiangyang city, Hubei province	78	32°00′ 56.49′′	112◦09′ 59.91′′	40	10	10	10	10	
	Guanmenshan Town, Shennongjia Forestry District, Hubei Province	1247	31°26′ 55.95′′	110°23′ 89.11′′	12	3	3	3	3	
Hunan	Jingya Forest Farm, Cili County, Zhangjiajie City, Hunan Province	334	29°31′ 22.69′′	110°46′ 02.50′′	40	10	10	10	10	79
	Zhaiyang Township, Jishou City, Hunan Province	285	28°18′ 17.38′′	109°38′ 13.42′′	39	10	10	10	9	
Jiangxi	Yinhe Town, Luxi County, Pingxiang City, Jiangxi Province	150	27°41′ 55.96′′	114°05′ 49.77′′	39	11	9	9	9	39
Shaanxi	Jinjiahe Town, Lueyang County, Hanzhong City, Shaanxi Province	727	33°20′ 02.71′′	106°30′18.25′′	44	11	11	11	11	84
	Longling Town, Hanyin County, Ankang City, Shaanxi Province	443	32°54′11.78"	108°30′36.21"	40	10	10	10	10	
Xinjiang	Liushihu Village, Ürümqi City, Xinjiang Province	205	44°32′ 35.60′′	87°27′ 45.06′′	20	5	5	5	5	28
	Ziniquanzi Town, Fukang City, Xinjiang Province	513	44°02′ 35.60′′	88°35′ 38.32′′	8	2	2	2	2	

### FT-NIR spectra acquisition

The sample powder was scanned using a NIR spectrometer (PerkinElmer, USA) equipped with a diffuse reflectance accessory. Each sample was weighed (1.0 ± 0.05 g) with an electronic balance (Sartorius, Germany) and placed in a clean glass for scanning to avoid errors in anthropic factors. The scan range was 10,000-4000 cm^−1^, the resolution was 4 cm^−1^, and 32 scans were accumulated for each spectrum. The ordinate of the spectrum is the log (1/reflectance) of the reciprocal reflectance. Each sample was measured three times in parallel and the final average spectrum was obtained using the OMICA software that the ordinate was set as the absorbance. To eliminate the interference of air information, before each scan, the laboratory air (H_2_O and CO_2_) spectrum was recorded as the background and automatically subtracted. In addition, it is necessary to control experiment’s room temperature and humidity (25 °C/30% RH) to maintain the consistency of the experimental operating environment. Finally, the data processing was used by SIMCA-P+14.1 software to analyze the acquired spectral data for subsequent modeling.

### Exploratory analysis and hierarchical cluster analysis

Exploratory analysis is an initial approach to study raw clustering results with a subset of variables or the entire dataset. The data is transformed into a new coordinate system and the correlation between the sample and the variable, can be seen through the extracted principal components. Thus, the classification trend of the sample can be visually analyzed by selecting the first two principal components in PCA to explain most of the spectral variable information. Unlike PCA, HCA determines the difference between the data point of each category according to all the data points’ distances to determine the similarity between them. The two closest data or categories are combined to generate a clustering tree. They were clustered into one group for samples with similar chemical information, while samples with larger differences were divided into different groups based on the theory of phylogenetic clustering algorithm [22]. In this work, using the average original spectral information of each classified sample for different drying methods and regions. PCA is done by SIMCA14 software, and HCA is analyzed by Origin 2021 software. Different from the past, the HCA in the form of a circle was executed.

### Two-dimensional correlation spectroscopy spectra image acquisition

Traditional Chinese medicines have the characteristics of “multi-component, multi-target and multi-channel”. Since the signals of different chemical components overlap each other, it is difficult to extract the information of interest and its overall quality evaluation is difficult. Therefore, we still need to resort to chemometric tools to find useful spectral features. The one-dimensional spectrum has the disadvantages of peak overlap and inconspicuous characteristic peaks. In contrast, generalized 2DCOS is an effective technology to improve spectral resolution and resolve peak overlap issues, which is widely used in physics, chemistry, medicine, and other fields [23, 24].

According to the theory of Noda, when measuring the equally spaced external perturbation *t* at n steps, the dynamic spectrum intensities are represented by the column vector *Y* at variable *v* [25]. The expression is as follows.

1 $$Y\left(v\right)=\left[\begin{array}{c}y\left(v,{t}_{1}\right)\\ y\left(v,{t}_{2}\right)\\ \begin{array}{c}y\left(v,{t}_{3}\right)\\ \vdots \\ y\left(v,{t}_{n}\right)\end{array}\end{array}\right]$$

Then, the synchronous ($\Phi$) and asynchronous ($\Psi$) two-dimensional correlation intensities between the different frequencies of *v*_1_ and *v*_2_ can be expressed as [26–28]:

2 $$\Phi \left({v}_{1},{v}_{2}\right)=\frac{1}{m-1}Y{\left({v}_{1}\right)}^{T}\cdot Y\left({v}_{2}\right)$$

3 $$\Psi \left({v}_{1},{v}_{2}\right)=\frac{1}{m-1} Y{\left({v}_{1}\right)}^{T}\cdot D\cdot Y\left({v}_{2}\right)$$

Then, the *D* in the above represents the *j*-th row and *k*-th column of the Hilbert-Noda transformation matrix, which is defined as follow:

4 $$ D_{{jk}}  = \left\{ \begin{gathered}   0,\quad j = k \hfill \\   \frac{1}{{\pi \left( {k - j} \right)}},\quad j \ne k \hfill \\  \end{gathered}  \right. $$

What’s more, $\mathrm{W}$ is the integrated two dimensional correlation intensity, and the integrated 2DCOS intensity between the different frequencies *v*_1_ and *v*_2_ is obtained by multiply ($\Phi \left({v}_{1},{v}_{2}\right)$ and $\Psi \left({v}_{1},{v}_{2}\right)$ according to the above description, the formula is shown as:

5 $$\mathrm{W}\left({v}_{1},{v}_{2}\right)=\Phi \left({v}_{1},{v}_{2}\right)\cdot \Psi \left({v}_{1},{v}_{2}\right)$$

In this work, matrix *Y* (m × n) contains two spectral data (m = 2): the first is the average FT-NIR spectrum and the second is the *i*-th FT-NIR spectrum about each drying methods or regions of EULs. The synchronous 2DCOS, asynchronous 2DCOS, and i2DCOS spectra of the *i*-th sample of each drying method or region can be obtained through formulas (2), (3), and (5), respectively. All samples respectively converted into spectral data by SIMCA 14.1. Before building the deep learning model, each type of 2DCOS image was divided into three parts. 60% samples of each drying methods or regions were selected for modeling as train set. Then, 30% samples were chosen by the Kennard-stone algorithm as test set. The remaining 10% samples were used for the external verification set.

### Establishment of PLS-DA model

PLS-DA is a linear supervised classification method established based on the standard PLS regression algorithm. Finding the variable with the largest covariance with the classification matrix (Y) from the variable matrix (X), where Y is divided into two categories, Y = 1 represents that sample belongs to a specific classification, Y = 0 represents that the sample does not belong to a specific classification. Finally, the probability is obtained that each sample is classified into each category. The algorithm could explain the small number of sample observations and reduce the effect of multicollinearity between the samples. Stability evaluation of the PLS-DA model based on two parameters are cross-validation root mean squared error (RMSECV) and root mean square error of estimation (RMSEE). The smaller the value indicates that the stability of this model is better, and the predictive ability is better. In addition, the performance of the classification model is evaluated by combining specificity, sensitivity, and accuracy. The closer the value is to 1, the better the performance of the classification model. The detailed expression of sensitivity, specificity, and accuracy is calculated using true positive (TP), false positive (FP), true negative (TN), and false negative (FN), which are as follows:

6 $$Sensitivity\left(SEN\right)=\frac{TP}{\left(TP+FN\right)}$$

7 $$Specificity\left(SPE\right)=\frac{TN}{\left(TN+FP\right)}$$

8 $$Accuracy\left(ACC\right)=\frac{TN+TP}{TN+TP+FP+FN}$$

Besides, receiver operating characteristic (ROC), is graphically represented by using group probabilities obtained from PLS-DA analysis. The area under the curve (AUC) of ROC, as an indicator of predictive performance, the value close to 1 demonstrates high predictive power of the classification model [29]. 200 times of iterations were performed by SIMCA 14.1 software to test the robustness and fitting degree of the PLS-DA model based on the parameters of R^2^-intercept and Q^2^-intercept. In this study, we tried to establish the PLS-DA model based on FT-NIR spectral data of EULs with different drying methods and regions. Using the Kennard-stone algorithm to divide the data set to improve the model recognition ability and avoid the irreproducibility of random selection. Data sets of different drying methods include 70% training set (374) and 30% test set (160), and the datasets of different regions are divided into 70% training set (372) and 30% test set (162) for the PLS-DA model.

### ResNet model establishment

ResNet is understood as performing “feature learning” or “representation learning”. The Anaconda and Python were installed as the data processing hardware platform and we selected Amazon’s MXNet as the ResNet framework. In addition, the MxBoard was installed for training process visualization. ResNet, as an excellent CNN, could overcome the problem of network degradation with increasing depth by residual block as displayed in Additional file 1: Figure S1. The residual block superimposing y = x layers (shortcut connections, also called as identity mappings).

**Fig. 2:**
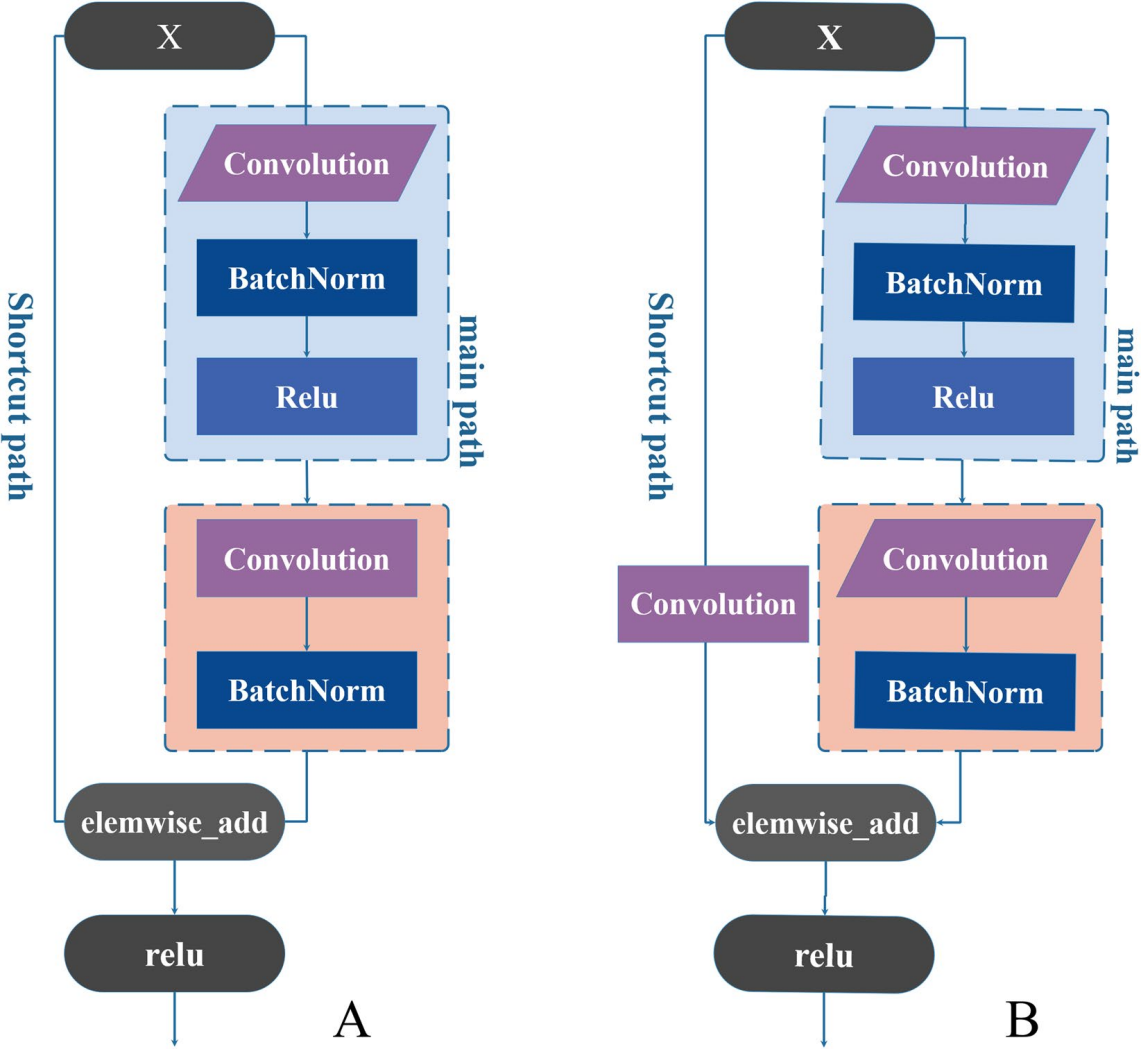
**A** Schematic diagram of identity block; **B** Schematic diagram of conv block

The ResNet network ensures the integrity of information. Therefore, the learning objective is F(x) = *H(x) −x*, which is simplified for the entire data processing. According to the dimensions of input and output whether were the same, it’s used the identity residual block (Identity block) and the convolution residual block (Conv block) to build the model. If the dimensions of output *F(x)* are consistent with that of input, the model was established depending on the identity block, which is displayed in Fig. 2A. if the dimensions of output *F(x)* are in conformity with the input *x*, the shortcut path of a convolutional layer with a convolution kernel size (1 × 1) is added, this structure is called conv block which is displayed in Fig. 2B.

In this study, we established a 12-layer ResNet model with the two types of residual blocks above whose input data are synchronous 2DCOS, asynchronous 2DCOS, and i2DCOS spectral images. Furthermore, the input data is passed through a layer of convolution operation, followed by BatchNorm normalization and Relu nonlinear activation. Then, four identity blocks and two conv blocks were used to extract features. Finally, the global average pooling, the flatten layer, and the full connection layer were executed that each layer functions differently in processed data.

## Results and discussion

### Analysis of FT-NIR spectra

**Fig. 3:**
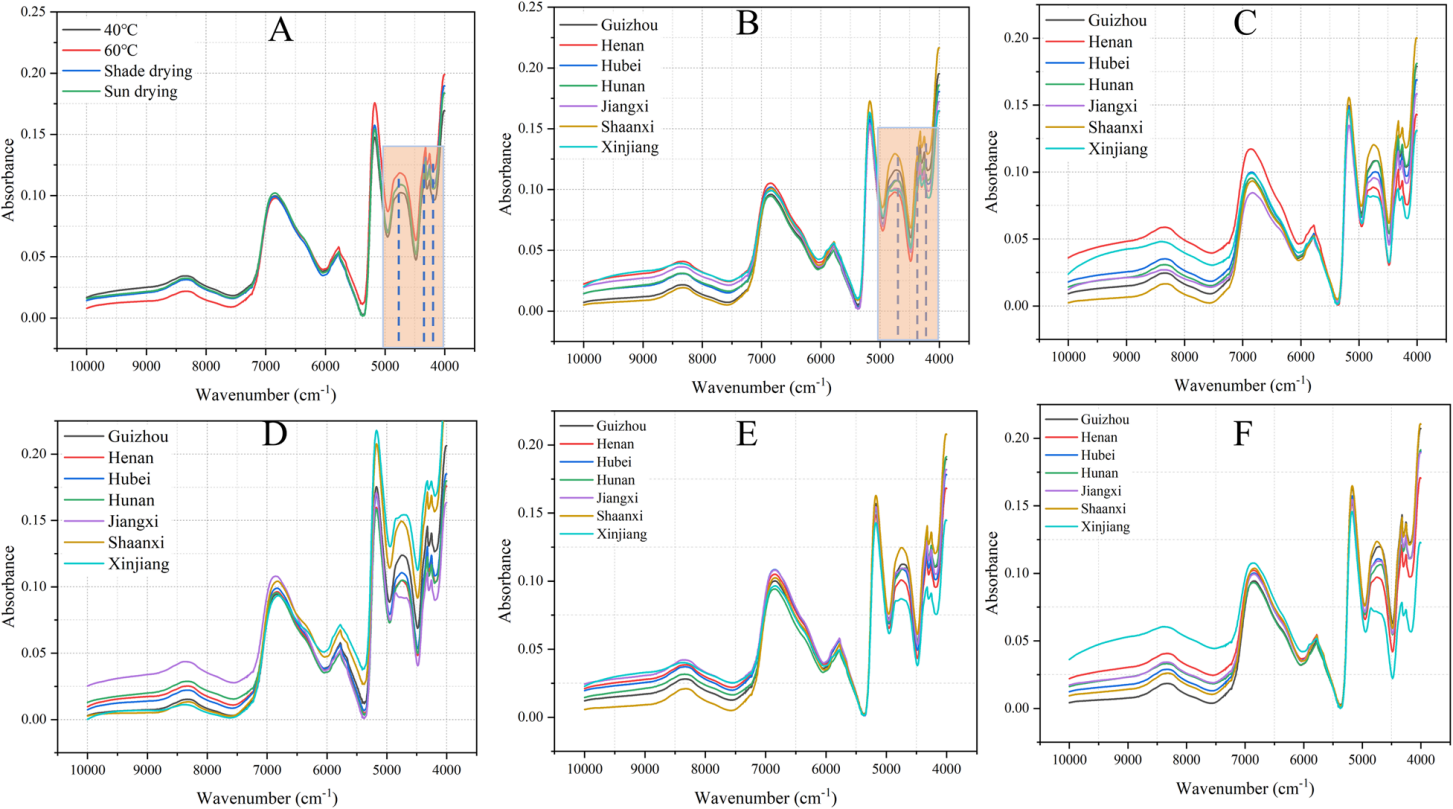
Original spectrum of different drying methods **A** and regions **B** for *E. ulmoides* leaves based on FT-NIR. **C**, **D**, **E**, **F**: different regions of 40℃, 60℃, sun drying, and shade drying, respectively

In order to reflect the originality of the data, the FT-NIR spectral data was obtained by MATLAB 14.1 only performed “automatic baseline correction” on it through OMICA software. All FT-NIR averaged spectra were shown in Fig. 3, including Figs. 3A, B, representing the average spectrum of different drying methods and seven regions. Figures 3C–F showed the average spectrum of seven regions for each processing method as follow: 40 °C, 60 °C, sun drying, and shade drying. From a comprehensive perspective, each FT-NIR spectrogram had wavenumbers at 8300 cm^−1^, which connected with the second overtone of C-H stretching vibrations of the CH_3_ and CH_2_ groups. The wideband at 6881 cm^−1^ was the first overtone of O–H stretching. The weak absorbance at 5775 cm^−1^ was related to C-H stretching of R-OHCH_3_, whereas an obvious peak at 5172 cm^−1^ was stretching of O-H and OH deformation of H_2_O. Moreover, the complex absorption peaks in the 5000-4000 cm^−1^ range, were C-H stretching and deformation group frequencies of polysaccharides and the C = O group frequencies and C-H stretching of carbohydrates.

The averaged spectra of four drying methods were exhibited in Fig. 3A. Taking 7000 cm^−1^ as the limit, the wave number was in the range of 10,000-7000 cm^−1^, the absorption peaks of the four drying methods have similar trends, but the absorption intensities were not consistent and the absorption peak intensity of the sample from high to low was 40℃ > sun drying > shade drying > 60℃. On the other hand, when the wave number was in the range of 6000-4000 cm^−1^, the absorption peak intensity was the largest at 60℃, followed by the spectrum of shade drying, sun drying, and 40℃. Among them, the changing trend and absorption intensity of the absorption peaks of sun drying and 40℃ were the same, which indicated that the chemical composition content in these two drying methods was similar. The averaged spectra of seven geographical regions were also exhibited in Fig. 3B. In the range of 10,000-7000 cm^−1^, the absorption peaks of different regions were Guizhou, Xinjiang, Jiangxi, Hunan, Hubei, and Shaanxi in descending order. However, in the range of 5350–4000 cm^−1^, the absorption intensity of Shaanxi is the highest, which indicated that the samples from Shaanxi have strong absorption signals in this wavenumber range. From Fig. 3C–F, spectral changes of samples from different regions based on different drying methods were different. The wavenumber was in the range of 5000-4000 cm^−1^, and the absorption peak intensity of the Shaanxi sample based on drying was significantly higher than that of other regions.

In all results, the spectra of EULs in different drying methods and different regions had the same trend and the peaks appear in similar positions. It meant that the chemical components contained in EULs from different regions were similar, but the absorbance difference was obvious, which represented the content of chemical components was different between different drying methods and different regions. Besides, the differences in absorption intensity and peak shape in different drying methods (Fig. 3A) were much lower than those in different regions, which demonstrated that the differences within individuals may be greater than the differences between drying methods, and it’s easier to identify regions than drying methods. Nevertheless, this conclusion needs further research and modeling analysis to support it.

### Exploratory analysis and hierarchical cluster analysis

**Fig. 4:**
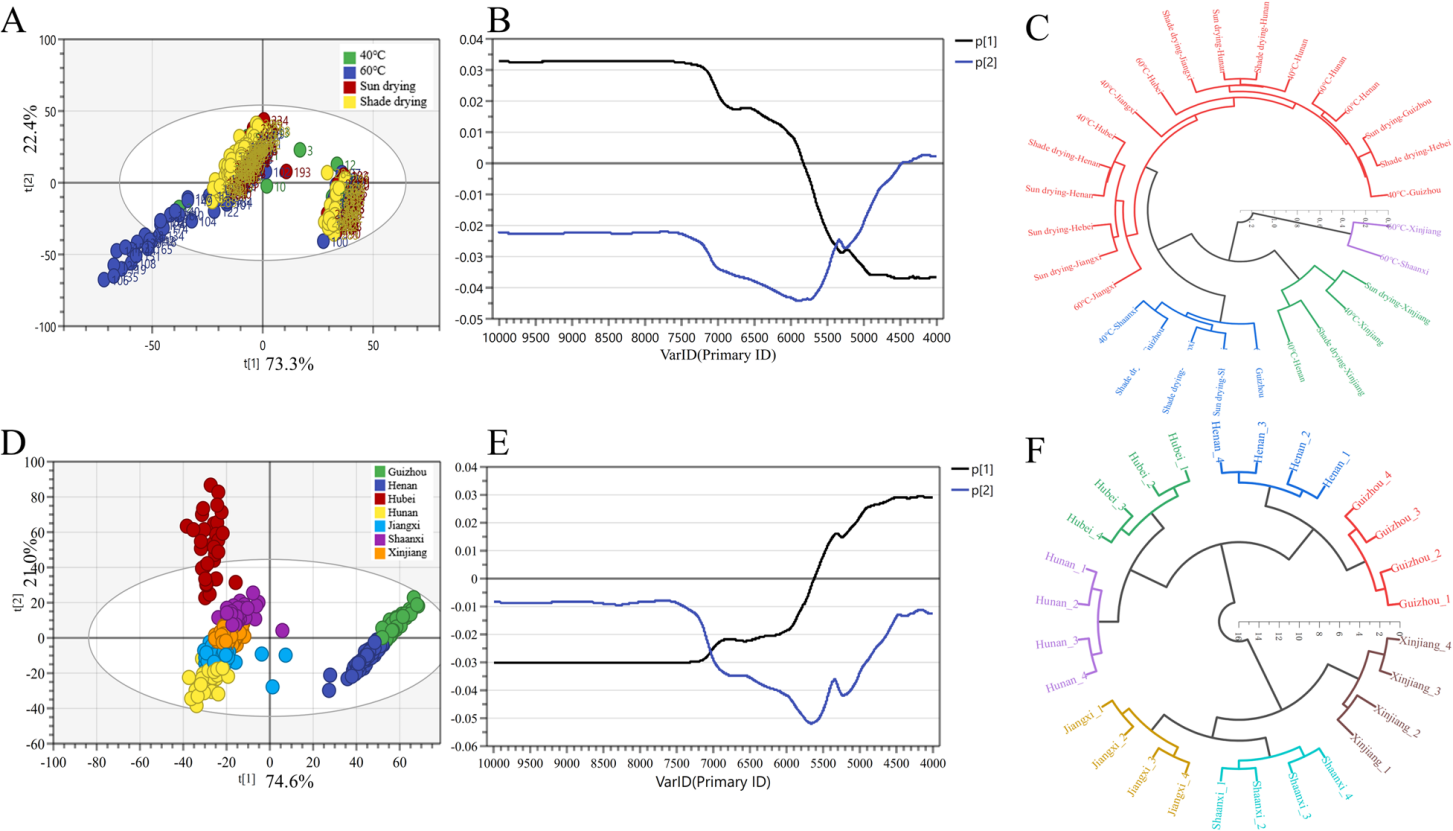
Score plots (**A** and **D**), loading plots (**B** and **E**) of PCA and HCA (**C** and **F**) for different drying methods and regions

The result of the PCA score plot based on the FT-NIR spectra data for different drying methods was shown in Fig. 4A. Analysis from a general point of view, these samples of different drying methods were difficult to distinguish with FT-NIR spectral characteristics. Some samples of 60 °C drying were separated from the other processed methods, some 60 °C samples also were covered by other sample points, and the rest of the samples were gathered together. It indicated that the chemical information of EULs samples between different drying methods was similar. The first two components of principal component 1 (PC1) and principal component 2 (PC2) accounted for 95.7% of the total variance (73.3% and 22.4%), revealing that most information of original data was preserved. The result of the PCA score plot based on the FT-NIR data for different regions was displayed in Fig. 4D. Seven regions were separated according to different categories, which was significant differences in chemical information between different regions.

According to the loading plot of the two principal components for different drying methods and regions as shown in Fig. 4B, E, respectively. PC1 and PC2 stressed almost all spectral signals, showing a profile close to those of the original FT-NIR spectra. The variables positively influencing the samples’ distribution at 10,000-5800 cm^−1^ for PC1 and the variables negatively influencing samples distribution were the 5800-4000 and 10,000-4600 cm^−1^ for PC1 and PC2 in Fig. 4B, respectively. Considering the consistency of loading values and PCs in Fig. 4E, the loading score of spectral variables at 10,000-5700 cm^−1^ and 10,000-4000 cm^−1^ drop to negative values for PC1 and PC2, respectively. However, spectral variables and PC1 had a positive effect in the range 5700-4000 cm^−1^.

In order to evaluate the similarity between different classified samples, HCA was established. This technology is designed to create groups that maximize internal cohesion and maximize external separation. The results of HCA for different drying methods and regions were shown in Fig. 4C, F. It is unsatisfactory that the clustering effect of different drying methods is chaotic, yet all samples were successfully clustered into seven parts according to category of regions. It shows that the EULs samples from different regions have obvious differences. This result is consistent with analysis of FT-NIR spectra and PCA.

### Analysis of 2DCOS images

**Fig. 5:**
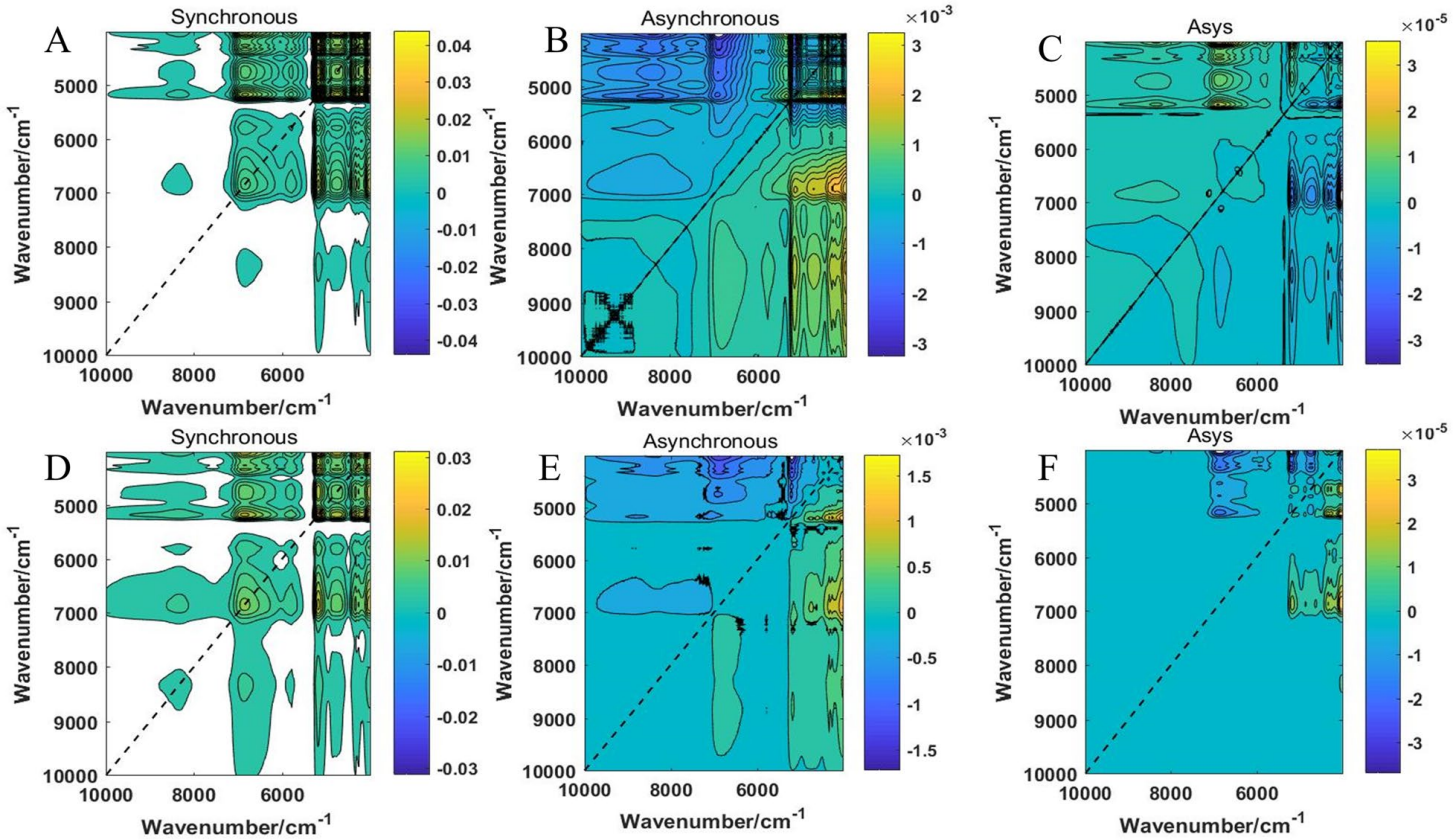
Synchronous, asynchronous, and integrated 2DCOS of different drying methods (**A**, **B**, **C**) and regions (**D**, **E**, **F**) for *E. ulmoides* leaves

Synchronous, asynchronous, and integrated 2DCOS images were shown in Fig. 5. 2DCOS (synchronous, asynchronous, or integrated) were obtained by MATLAB 14.1 software based on the full spectrum of 10,001–4000 cm^−1^. It contained more important information on molecular structure, improving the resolution of 1D spectroscopy, which could be used to identify and study the interactions between substances or groups [25]. The peaks located on the main diagonal in the synchronous 2DCOS and were called auto-peaks, which reflected the sensitivity of the changes of the related spectra in different regions for each chemical group in the sample to perturbation variables. The auto-peaks are more sensitive to perturbing variables, the auto-peaks intensity will be stronger. The non-main diagonal peaks were called cross peaks, and reflected the relativity in the intensity vibrations corresponding to their frequencies [29]. Automatic peaks were always positive, but cross peaks could be positive or negative. A cross peak on a 2DCOS was positive if two peaks of different wavenumbers decrease or increase at the same time, and it was negative otherwise. Asynchronous 2DCOS, used to characterize the degree of difference between two spectral signals. Their value reaches a maximum or minimum value when two spectral signals are orthogonal to each other. Its value was zero when the two dynamic signals are in phase or out of phase. The asynchronous 2DCOS was antisymmetric on both sides of the diagonal that consisted with entirely of cross peaks and had no automatic peaks. In general, if *v*_*1*_ changes strongly before *v*_*2*_, a positive peak appears; if *v*_*1*_ changes strongly after *v*_*2*_, a negative peak appears. In this study, Figs. 5A–C represented the synchronous, asynchronous, and integrated 2DCOS images of different drying methods of EULs, respectively. Figures 5D–F represented the synchronous, asynchronous, and integrated 2DCOS images of different regions of EULs. Compared asynchronous and integrated 2D correlation spectroscopy, the peaks value of the synchronous 2DCOS were clear. The feature information was relatively obvious, revealing that synchronized 2DCOS was suitable for intuitive analysis of characteristic peaks. The auto-peaks of synchronous 2DCOS in Figs. 5A, D were mainly concentrated in the wavenumber range of 7250-4000 cm^−1^. It was related to substances such as water and carbohydrate that this result was consistent to “analysis of  FT-NIR spectra”. The information of asynchronous and integrated 2DCOS images was fragmented with less spectral information, it was not suitable for identification. In addition, the 2DCOS images of different drying methods and regions were shown in Additional file 1: Figures S2 and S3. The traditional Chinese medicine of EULs is a complex mixed system, which contains a variety of chemical components and is affected by various factors of the natural environment. Therefore, the difference and intensity variation of automatic peak and cross peaks in EULs’ samples of different drying methods and regions to achieve the purpose of identification. It is necessary to further use the discriminative model for identification and evaluation.

### Discrimination of *E. ulmoides* leaves with the different drying methods

#### PLS-DA model

**Fig. 6:**
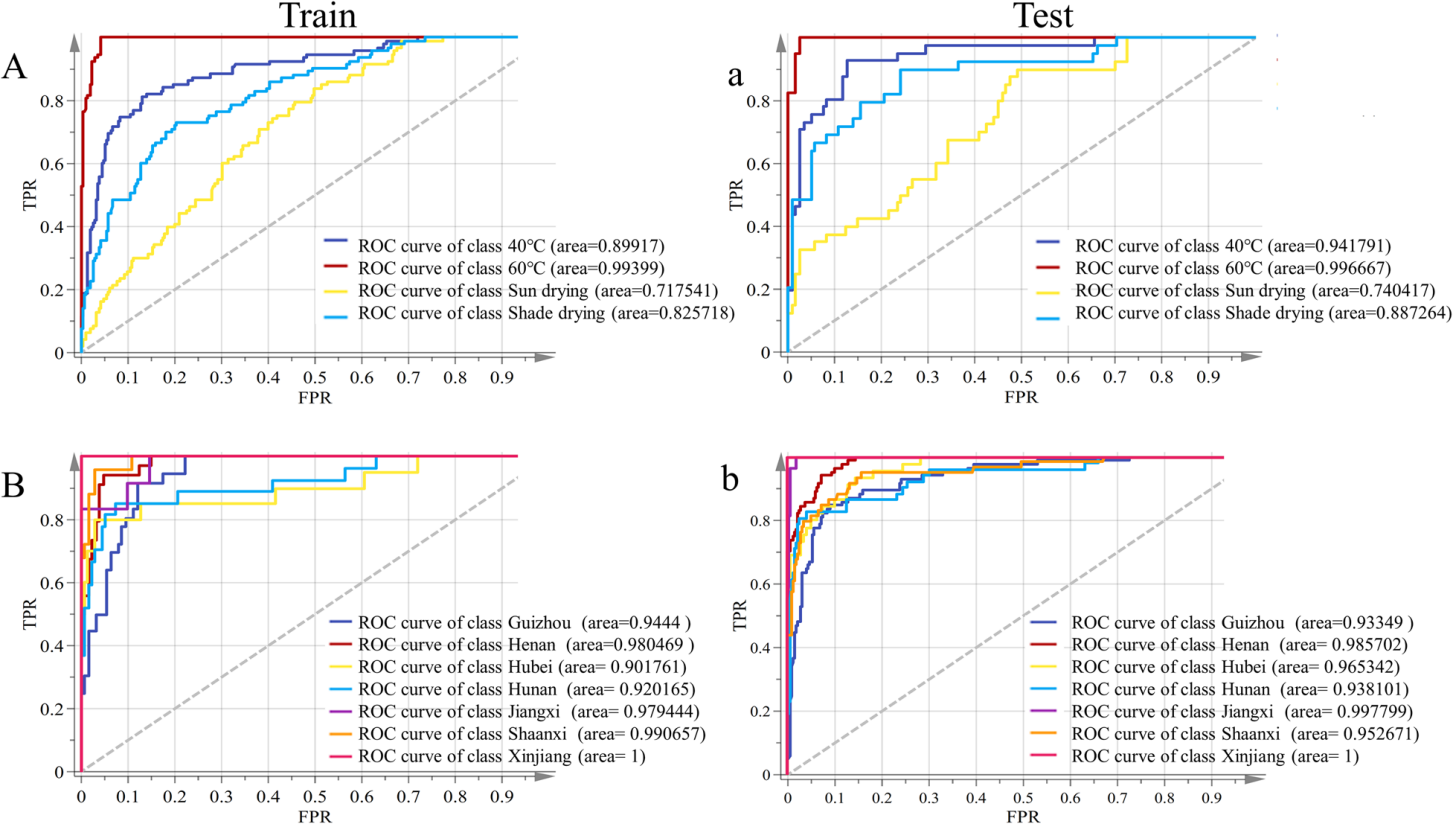
Receiver operating characteristic (ROC) curves with the area under curve (AUC) of different drying methods (**A** and **a**) and different regions (**B** and **b**)

Identifying individuals of different groups based on their spectral characteristics is one of the main tasks of chemometrics research. It’s best to use a fixed method and see how that method handles it to get a head start when analyzing two spectral data sources. In this study, the PLSDA model was firstly used to evaluate the differences of different drying methods and regions of EULs, and the variable characteristics of spectral sets were comprehensively studied. Based on the spectral data of different dry methods, the PLS-DA model was established that the best number of latent variables was 7 as displayed in Additional file 1: Figure S4 A. Besides, a slightly higher RMSEE (0.35066) and RMSECV (0.35962) were obtained, but within the allowable value of the model parameters. The ROC curve reaches the upper left corner, indicating that the specificity can be good without reducing the sensitivity. For a good model, the AUC, sensitivity, and specificity values are all between 0 and 1 and should be close to 1. More information on the sensitivity and specificity of the model can be found from references [30]. Figure 6A displayed ROC curves, and each graph expressed the sensitivity and specificity of the model as a function of the selected threshold. The confusion matrix for different dry methods of EULs based on PLS-DA was shown in Additional file 1: Table S1. The parameters result in sensitivity, specificity, accuracy, and AUC value for different drying methods as shown in Table 2. The results displayed that a sensitivity (0.9677, 0.9750), specificity (0.9609, 0.9664), accuracy (0.9677, 0.9625), and AUC value (0.9934, 0.9967) of train and test set based on 60℃ data were higher than other samples of other categories. It implied that samples processed by this drying method could lead to significant differences in chemical composition or content between samples. At the same time, only three out of 93 samples and one out of 40 samples for train set and test set with 60℃ drying methods were misclassified, respectively, and the correct classification rates reached 97.5%. The least ideal parameter values were the sun drying samples. Although the specificity values were both above 0.9300, the sensitivity of the train set and test set were only 0.2796 and 0.6667, respectively. The accuracy and AUC values were lower than 0.9000, and indicated that the sample separation effect is not good. Additionally, the response permutation test of 200 times (Y scrambling) was shown in Additional file 1: Figure S4 a, which revealed no overfitting with R^2^ of R^2^Y-intercept of −0.0034, Q^2^-intercept of -0.0834, showing that the method has a good identify ability and no over-overlap phenomenon.

**Table 2:** The classification parameters of PLS-DA established for different regions and drying methods

Styles	Classes	Train set				Test set			
		SEN	SPE	ACC	ROC	SEN	SPE	ACC	ROC
different regions	Guizhou	0.8117	0.7124	0.8978	0.9444	0.9722	0.8880	0.9012	0.9335
	Henan	0.9405	0.7446	0.9570	0.9805	0.8857	0.9685	0.9506	0.9852
	Hubei	0.7556	0.8844	0.9489	0.9018	0.7368	0.9720	0.9444	0.9653
	Hunan	0.7885	0.8414	0.9516	0.9202	0.6296	0.1000	0.9383	0.9358
	Jiangxi	0.8519	0.9167	0.9785	0.9794	0.5000	0.1000	0.9630	09,978
	Shaanxi	0.7458	0.8118	0.9301	0.9907	0.1000	0.9648	0.1000	0.9527
	Xinjiang	0.9500	0.8925	0.9167	0.1000	0.8750	0.1000	0.9938	0.1000
Different processing	40°C	0.8000	0.9315	0.8279	0.8991	0.8537	0.9160	0.9000	0.9418
	60°C	0.9677	0.9609	0.9677	0.9934	0.9750	0.9664	0.9625	0.9967
	Sun drying	0.2796	0.9395	0.7754	0.7175	0.6667	0.9308	0.8563	0.7404
	Shade drying	0.6667	0.8398	0.8011	0.8257	0.7949	0.8678	0.8500	0.8873

*SEN* Sensitivity, *SPE* specificity, *ACC* Accuracy, *ROC* Receiver Operating Characteristic

#### ResNet model

**Fig. 7:**
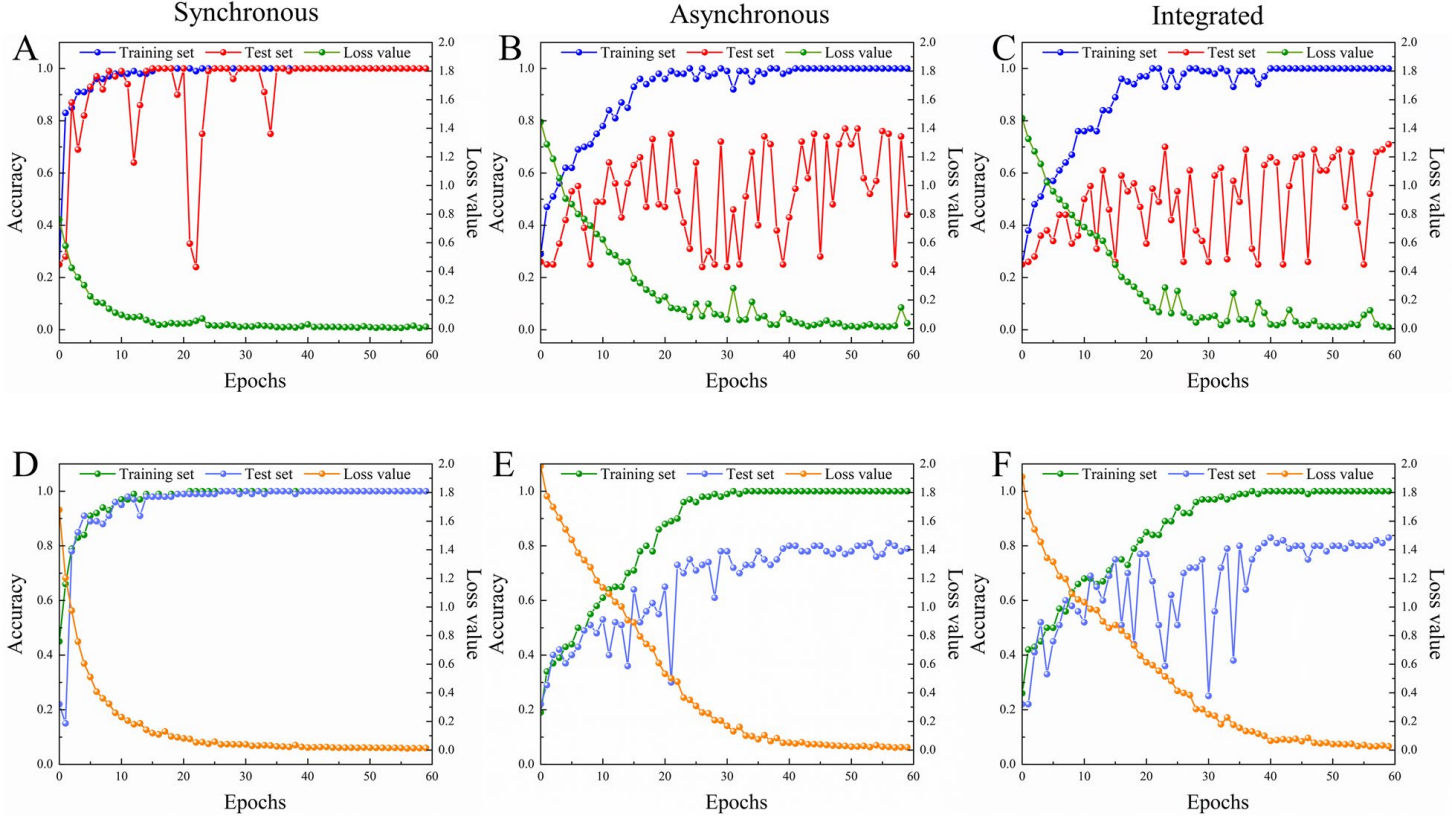
The accuracy curves and cross-entropy cost function of synchronous, asynchronous, integrated 2DCOS spectra model for different drying methods (**A**, **B**, **C**) and regions (**D**, **E**, **F**)

Results of synchronous, asynchronous, and integrated 2DCOS images based on “Analysis of 2DCOS images”, ResNet models were established as displayed in Fig. 7. The cross-entropy cost function curves and accuracy curves are the parameters to evaluate the model. Among them, the cross-entropy loss function is applied to account for the convergence effect of the model that the closer its value is to zero, the better the convergence of this model. The recognition ability of the model is evaluated by the accuracy curves of the training set and the test set. The value is closer to 1, representing that the identify ability of the model is stronger.

**Fig. 8:**
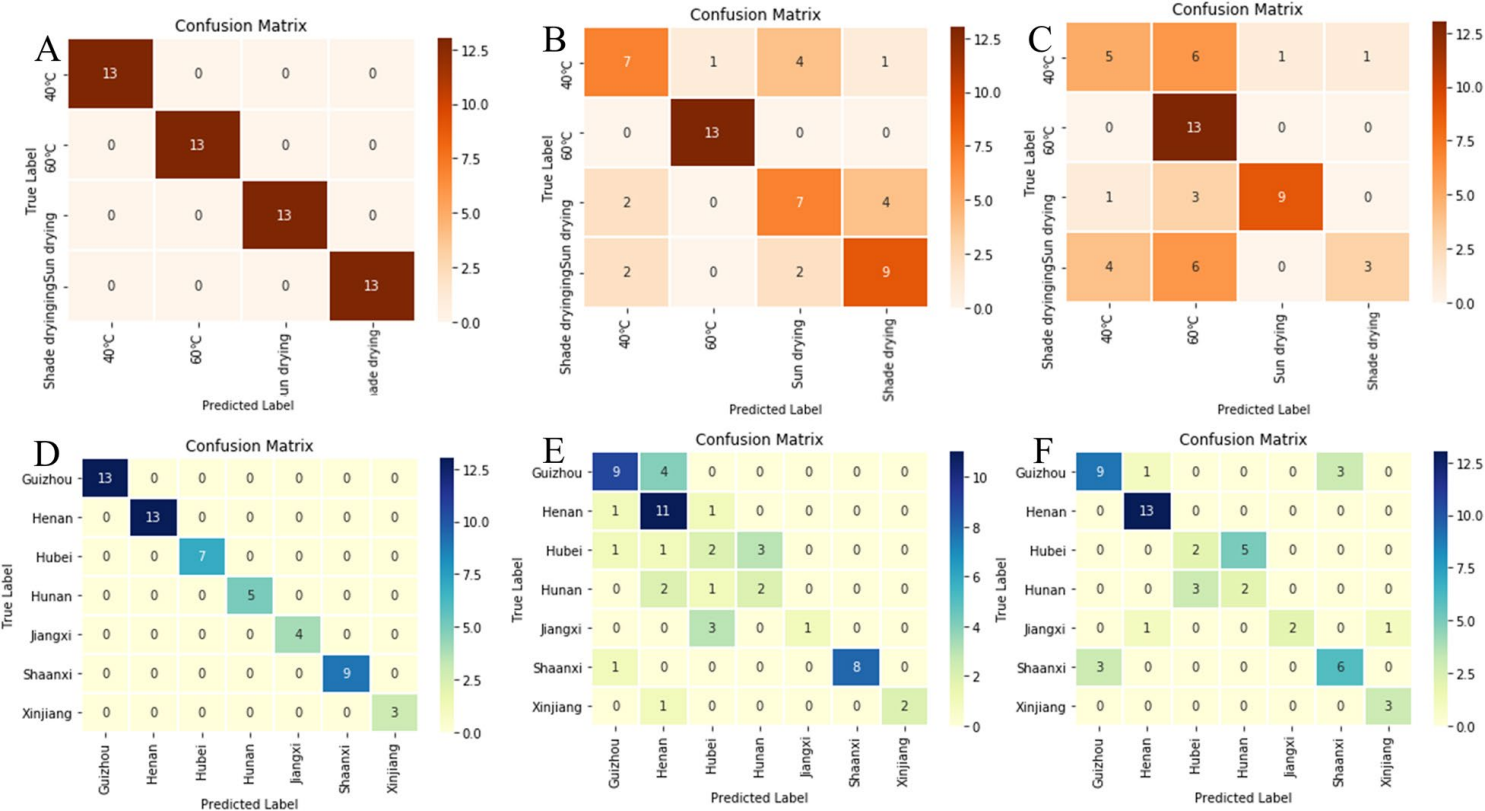
The confusion matrix of synchronous (**A** and **D**), asynchronous (**B** and **E**), integrated (**C** and **F**) 2DCOS spectra model for different drying methods (**A**, **B**, **C**) and regions (**D**, **E**, **F**)

In this work, when the number of epochs is 37, the accuracy of both the training set and the test set reached 100%, and the loss value was 0.0214 and tended to be flat from Fig. 7A. It showed that this model has strong generalization and stability ability and could be used to identify the different drying methods. Compared with the model of the synchronous 2DCOS, the results of asynchronous and integrated 2DCOS model were poor that were not suitable for identifying the different drying methods of these EULs. The color information of the synchronous 2DCOS images may be rich than the asynchronous spectrum, and the line information of auto-peak and cross-peak is clear. Even though the number of epochs was 42, the performance of the training set reached 100%. However, when the number of epochs was 60, the accuracy of the test set did not reach 100% and fluctuates widely as shown in Fig. 7B, C, indicating that the accuracy and generalization ability of the model were all poor. The reason for this phenomenon may be caused by the similar spectral information or less characteristic spectral information obtained by asynchronous 2DCOS images of samples with different drying methods. In addition, the model established above was used to classify the external validation set, and the classification results of the external validation set for different parts are shown in the confusion matrix of the synchronous, asynchronous, and integrated 2DCOS in Fig. 8A–C, respectively. Figure 8A showed that all 52 samples of the drying methods were correctly identified. 12 samples out of 52 were misclassified as shown in Fig. 8B. Among them, the samples based on 60 °C were completely classified correctly, while the samples of other categories were all wrongly classified. four samples were misclassified based on shade drying samples, six samples were misclassified for both 40 °C and sun drying samples, and only one sample at 40 °C was misclassified in the 60 °C category of all misclassified samples. Figure 8C also had the same trend that 13 samples based on 60 °C were completely classified correctly. This result is consistent with spectral feature information and PCA conclusion. This may be the significant change in the content of chemical components in the samples after drying at 60 °C, and there is a similarity in the content of chemical components between samples after other drying methods.

### Discrimination of *E. ulmoides* leaves with different collection regions

#### PLS-DA model

The identify significant differences between groups of surveyed samples was executed by the PLS-DA model when studying differences between internal partial samples in different regions, which represented the total variance in the spectral information (R^2^ = 0.536 and Q^2^ = 0.387). The best number of latent variables (LVs: 22) and permutation test of 200 times also showed that the model was rigorously set up with no overlap as displayed in Additional file 1: Figures S4 B and b. The two parameters of RMSEE and RMSECV were 0.23794 and 0.26984, respectively, indicating that the classification model performs was good and could be used for identification of different regions. The seven groups of Henan, Hubei, Hunan, Jiangxi, Shaanxi, and Xinjiang had good discrimination results that the accuracy of the train and test set were 0.8978 and 0.9012 as shown in Table 2, respectively. The same trend of AUC values, AUC values for all different regions were above 0.9018 in Figs. 6B, b. These results showed that the comprehensive spectral information could distinguish EULs from different regions, revealing that the it’s different from chemical properties of different regions samples. This model exhibited relatively slightly high sensitivity and specificity for the train set, especially for Jiangxi and Xinjiang, both values were above 0.8519. Regrettably, the sensitivity values in Hubei, Hunan, and Jiangxi were from 0.5000 to 0.7368. The sensitivity and specificity of the test sets from other regions were both above 0.875. At the same time, the confusion matrix of different regions was shown in Additional file 1: Table S2. The classification results of the test set from Guizhou, Shaanxi, and Xinjiang provinces were good, with only one misclassified or completely correctly classified. Overall, the results for all parameter values of PLS-DA model in Xinjiang were excellent. These results indicated that there are significant differences in chemical information between samples from three regions and samples from other regions, this result was consistent with “Analysis of FT-NIR spectra”.

#### ResNet model

The ResNet models were established that weight attenuation coefficient λ was 0.0001, the learning rate was 0.01, and batch_size was 16. The accuracy curve and the cross-entropy cost function curve were generated by Mxboard, where the smoothing parameter of the curve was 0.6. The regions and drying methods discrimination strategy of EULs based on ResNet as shown in Additional file 1: Figure S5. The accuracy curves and the cross-entropy cost function of synchronous, asynchronous, and integrated 2DCOS were displayed in Fig. 7. Figure 7D was the cross-entropy cost function of the precision curve of the synchronous 2DCOS spectrum. We could see that the epoch was 27, the accuracy of the training set and test set was 100%, and the loss value was close to zero. The results demonstrated that the synchronous 2DCOS model had a good ability to discriminate the regions. The accuracy curve and cross-entropy cost function of the asynchronous 2DCOS model was given as shown in Fig. 7E. It could be seen that the epoch was 34, the accuracy of the train set was 100% and the test set was 74.6%. Although the value of the test set fluctuates as the number of epochs increases, it never reaches 100%. Similarly, when the number of epochs was 39, the accuracy of the train set and test set was 100% and 84%, respectively, and the loss value was 0.059. Even with the increase in the number of epochs, the accuracy of the training set does not increase in Fig. 7F. It showed that there were no good models of asynchronous and integrated 2DCOS for geographical identification of EULs.

Moreover, the 54 external validation of 2DCOS images (synchronous 2DCOS asynchronous 2DCOS, and integrated 2DCOS) were established. The confusion matrix of synchronous 2DCOS asynchronous 2DCOS, and integrated 2DCOS models were shown in Fig. 8D–F, respectively. Figure 8D showed that all samples were correctly classified with 100% accuracy for geographical identification. For Fig. 8E, a total of 35 samples were correctly classified, accounting for 64.8% of the total samples. Even if the generalization ability of the model was not very good, the regions of Henan and Shaanxi were classified correctly, accounting for 81.8% and 87.5%, respectively. Surprisingly, 36 samples were correctly classified in the confusion matrix of integrated 2DCOS, and the 13 samples from Henan and 3 samples from Xinjiang were completely correctly classified in Fig. 8F. Therefore, the synchronous 2DCOS images model of EULs for geographical identification had the strongest generalization ability. However, three models had good identification ability for the samples of EULs from Henan in the three models of synchronous 2DCOS asynchronous 2DCOS, and integrated 2DCOS. To sum up, the spectral information in the 2DCOS images of the samples in Henan, Shaanxi, and Xinjiang provinces might be very different from other provinces, so that samples could be classified correctly even when the generalization ability of the model was not very good. However, this requires more sample size to obtain the qualitative and quantitative experimental data to support this conclusion.

#### Combined with climate data analysis

The origin and growth environment of TCM are important, which greatly affects the efficacy of TCM. As a major factor, ecological environmental factors become a research hotspot, affect the quality of Chinese medicinal materials and the source of the formation of authentic medicinal materials. EULs like a warm and humid climate and a sunny environment and are resistant to severe cold. It can be cultivated in most areas of my country and has strong adaptability. There is no strict selection of soil, but the soil layer of deep, loose, fertile, moist, and well-drained loam is best. The growth rate of the *E. eucommia* tree is relatively slow in the juvenile period, and the fast-growing period appears in 7–20 years. It grows place more in low mountains, valleys, or low-slope sparse forests.

**Fig. 9:**
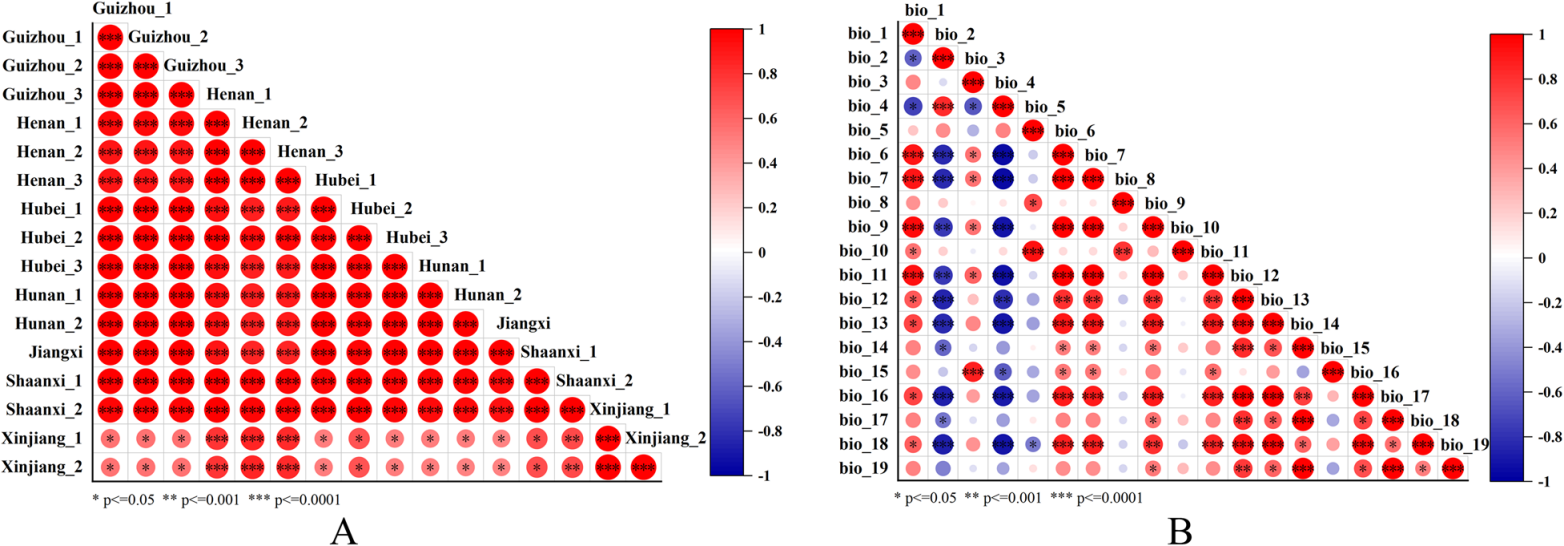
Correlation plots of 16 regions (**a**) and 19 climatic data (**b**) based on the FT-NIR spectra spectrum

The bioclimatic indicators were selected from the world climate-global climate database (WorldClim 1.4, http://worldclim.org/). In this study, the climate data such as average temperature, precipitation, solar radiation, maximum temperature, and minimum temperature were analyzed and evaluated. The 19 bioclimatic indicators of environmental indicators were listed in Additional file 1: Table S3. The distribution of average climatic data of 16 regions were showed in Additional file 1: Figure S6, the result displayed that the annual average temperature of six provinces was in the range of 9–16 ℃, while Xinjiang showed a significant difference from other provinces, and the annual average temperature was around 7 ℃. The mean diurnal range was a range in 7–12 ℃, and the provinces with relatively high values were Xinjiang and Henan. This may be related to the high temperature during the day in summer. At night, due to the lack of ability to retain heat, the ground cools and dissipates heat very fast and the temperature drops rapidly. The result revealed a particularly large temperature difference between day and night. This result of the temperature difference in Xinjiang was large, which can also be confirmed from max temperature of warmest month and min temperature of coldest month. In addition, the order of annual precipitation from high to low is Jiangxi, Hunan, Hubei, Guizhou, Shaanxi, Henan, and Xinjiang. In general, the climate of Xinjiang is characterized by dryness, which highlighted the decrease in precipitation. Besides, correlation plots using 16 regions’ spectral data and 19 climatic data as variables were discussed and displayed in Fig. 9. Figure 9A was a correlation plot based on 16 regions. The samples from the 16 regions had slight differences, and the correlation coefficients were all greater than 0.9 except for Xinjiang, showed a highly correlated. The difference between regions of Xinjiang and others were relatively obvious, and the correlation coefficient was around 0.5 except for Xinjiang and Henan (the correlation coefficient was about 0.8). These results were consistent shows that two Xinjiang and the other 11 regions have significant differences, which may be related to the topography of Xinjiang that locate in high latitudes and is the only mid-temperate region of all sample collection areas, the result was consistent as this report [31]. But there was a slightly higher similarity between three regions in Henan. Figure 9B was a correlation plot based on 19 climatic data with the p-value falling below the 0.05, 0.001, and 0.0001 levels of significance. We found that different significance levels were set to present similar results as displayed in Additional file 1: Figure S7. The correlation y between 19 climate data was generally lower than that of different regions only a few climate data had a high correlation, while there were stronger associations of climate data. For example, bio_1 had a significant positive correlation with 6, 7, 9, etc., but bio_2 had a significant negative correlation with 6, 7, 9, 11, 12, 13, 16, 18, and bio_4 result was similar to bio_2 for these several climatic data. Correlation plots more intuitively show high correlations and differences between different regions or between the two, and there are also differences in climate data.

## Conclusion

In this study, different drying methods and regions of EULs were successfully discriminated by the original dataset of FT-NIR without any preprocessing using 2DCOS directly incorporating ResNet with chemometrics. Exploratory analysis methods and hierarchical clustering analysis results show that the clustering effect of samples from different drying methods is not good, while samples from different regions reflect a good separation effect according to different categories. Besides, PLS-DA and ResNet models were used for certificating the classification performance. ResNet can be applied as the best choice for identifying the different drying methods and geographical traceability based on synchronized 2DCOS images due to the excellent model demonstrating the characteristics of time-saving, high accuracy, and simple operation. For ordinary people, this model does not require artificial feature extraction or complex data processing, just knowing the input and output, which greatly reduces the computational complexity and operation process. Due to the spectral information characteristics and the two models, the EULs samples had significant differences between 60 ℃ drying and other drying methods. The EULs samples from Henan, Shaanxi, and Xinjiang were also different from other regions and listed as a “National Geographical Indication Protected Product”, which may be related to the local climatic conditions and geographical advantage as the main regions of *E. ulmoides* cultivation, especially Xinjiang. However, these results can provide qualitative explanations and scientific support for the application of EULs. The results exhibit that the FT-NIR spectral information combined with the chemometric method can be quickly, effectively, and non-destructively applied to identification of drying method and geographical traceability for EULs. Therefore, this work not only provides more comprehensive evidence of chemical information for the key supplement for quality evaluation of EULs, but also provides a novel reference for discrimination of drying methods and regions on this medicinal and edible plants.

## Supplementary Information

**Additional file 1: Table S1.** Confusion matrix for different drying methods of *E. ulmoides* leaves based on FT-NIR. **Table S2.** Confusion matrix for different regions of *E. ulmoides* leaves based on FT-NIR. **Table S3.** 19 bioclimatic indicators of environmental indicators in this study. **Figure S1.** The residual block of deep learning. **Figure S2.** The synchronous, asynchronous, and integrated 2DCOS of different drying methods for *E. ulmoides* leaves. **Figure S3.** The synchronous, asynchronous, and integrated 2DCOS of different regions for *E. ulmoides* leaves. **Figure S4.** The optimal number of latent variables and permutation test of 200 times for different drying methods (A and a) and regions (B and b) of *E. ulmoides* leaves based on PLS-DA model. **Figure S5.** The regions and drying methods discrimination strategy of *E. ulmoides* leaves based on ResNet. **Figure S6.** The distribution of average climatic data of 16 regions. **Figure S7.** The correlation plot based on 16 regions and 19 climatic data with the p-value falls below the 0.05 (A and D), 0.001 (B and E), and 0.0001 (C and F) level of significance.

## Data Availability

Data is available on request to the authors.

## Abbreviations

EULs: *Eucommia ulmoides* Leaves
FT-NIR: Fourier yransform near infrared spectroscopy
2DCOS: Two-dimensional correlation spectroscopy
PLS-DA: Partial least squares discriminant analysis
ResNet: Residual neural network
