# Supplementary material for: A method of two-dimensional correlation spectroscopy combined with residual neural network for comparison and differentiation of medicinal plants raw materials superior to traditional machine learning: a case study on Eucommia ulmoides leaves
Source: Plant Methods. 2022 Aug 13;18:102. doi: 10.1186/s13007-022-00935-6 (PMC9375363; doi:10.1186/s13007-022-00935-6)
Supplement: Supplementary file 1 — Additional file 1: Table S1. Confusion matrix for different drying methods of E. ulmoides leaves based on FT-NIR. Table S2. Confusion matrix for different regions of E. ulmoides leaves based on FT-NIR. Table S3. 19 bioclimatic indicators of environmental indicators in this study. Figure S1. The residual block of deep learning. Figure S2. The synchronous, asynchronous, and integrated 2DCOS of different drying methods for E. ulmoides leaves. Figure S3. The synchronous, asynchronous, and integrated 2DCOS of different regions for E. ulmoides leaves. Figure S4. The optimal number of latent variables and permutation test of 200 times for different drying methods (A and a) and regions (B and b) of E. ulmoides leaves based on PLS-DA model. Figure S5. The regions and drying methods discrimination strategy of E. ulmoides leaves based on ResNet. Figure S6. The distribution of average climatic data of 16 regions. Figure S7. The correlation plot based on 16 regions and 19 climatic data with the p-value falls below the 0.05 (A and D), 0.001 (B and E), and 0.0001 (C and F) level of significance. [file 13007_2022_935_MOESM1_ESM.docx]

A method of two-dimensional correlation spectroscopy combined with residual neural network for comparison and differentiation of medicinal plants raw materials superior to traditional machine learning: A case study on *Eucommia ulmoides* leaves

Lian Li^a, b^, Zhi Min Li^a,^ *, Yuan Zhong Wang^a,^ *

^a^*Medicinal Plants Research Institute, Yunnan Academy of Agricultural Sciences, Kunming, 650200, P. R. China.*

^b^*College of Traditional Chinese Medicine, Yunnan University of Chinese Medicine, Kunming, 650500, P. R. China.*

*Corresponding authors:

Zhi Min Li: 393891330@qq.com

Yuan Zhong Wang: [boletus@126.com](mailto:boletus@126.com)

Table S1. Confusion matrix for different drying methods of *E. ulmoides* leaves based on FT-NIR.

Table S2. Confusion matrix for different regions of *E. ulmoides* leaves based on FT-NIR.

Table S3. 19 bioclimatic indicators of environmental indicators in this study.

Figure S1. The residual block of deep learning.

Figure S2. The synchronous, asynchronous, and integrated 2DCOS of different drying methods for *E. ulmoides* leaves.

Figure S3. The synchronous, asynchronous, and integrated 2DCOS of different regions for *E. ulmoides* leaves.

Figure S4. The optimal number of latent variables and permutation test of 200 times for different drying methods (A and a) and regions (B and b) of *E. ulmoides* leaves based on PLS-DA model.

Figure S5. The regions and drying methods discrimination strategy of *E. ulmoides* leaves based on ResNet.

Figure S6. The distribution of average climatic data of 16 regions.

Figure S7. The correlation plot based on 16 regions and 19 climatic data with the p-value falls below the 0.05 (A and D), 0.001 (B and E), and 0.0001 (C and F) level of significance.

.

Table S1. Confusion matrix for different drying methods of *E. ulmoides* leaves based on FT-NIR.

| Train test | | | | | |
| --- | --- | --- | --- | --- | --- |
|  | 40℃ | 60℃ | Sun drying | Shade drying | **Correct** |
| 40℃ | 76 | 2 | 7 | 10 | 80% |
| 60℃ | 1 | 90 | 0 | 2 | 96.77% |
| Sun drying | 33 | 1 | 26 | 33 | 27.96% |
| Shade drying | 13 | 8 | 10 | 62 | 66.67% |
| Total | 123 | 101 | 43 | 107 | 67.91% |
| Test set | | | | | |
| 40℃ | 35 | 0 | 5 | 1 | 85.37% |
| 60℃ | 1 | 39 | 0 | 0 | 97.50% |
| Sun drying | 7 | 2 | 16 | 15 | 40% |
| Shade drying | 2 | 2 | 4 | 31 | 79.49% |
| Total | 45 | 43 | 25 | 47 | 75.63% |

Table S2. Confusion matrix for different regions of *E. ulmoides* leaves based on FT-NIR.

| Train set | | | | | | | | |
| --- | --- | --- | --- | --- | --- | --- | --- | --- |
|  | Guizhou | Henan | Hebei | Hunan | Jiangxi | Shaanxi | Xinjiang | **Correct** |
| Guizhou | 69 | 3 | 2 | 4 | 3 | 4 | 0 | 81.18% |
| Henan | 0 | 79 | 2 | 1 | 0 | 2 | 0 | 94.05% |
| Hebei | 2 | 4 | 34 | 2 | 0 | 3 | 0 | 75.56% |
| Hunan | 7 | 0 | 2 | 41 | 0 | 2 | 0 | 78.85% |
| Jiangxi | 4 | 0 | 0 | 0 | 23 | 0 | 0 | 85.19% |
| Shaanxi | 9 | 3 | 2 | 0 | 1 | 44 | 0 | 74.58% |
| Xinjiang | 0 | 1 | 0 | 0 | 0 | 0 | 19 | 95.00% |
| Total | 91 | 90 | 42 | 48 | 27 | 55 | 19 | 83.06% |
| Test set | | | | | | | | |
| Guizhou | 35 | 0 | 0 | 0 | 0 | 1 | 0 | 97.22% |
| Henan | 0 | 31 | 3 | 0 | 0 | 1 | 0 | 88.24% |
| Hebei | 1 | 2 | 14 | 0 | 0 | 2 | 0 | 70.00% |
| Hunan | 7 | 1 | 1 | 17 | 0 | 1 | 0 | 62.96% |
| Jiangxi | 6 | 0 | 0 | 0 | 6 | 0 | 0 | 50.00% |
| Shaanxi | 0 | 0 | 0 | 0 | 0 | 25 | 0 | 100.00% |
| Xinjiang | 0 | 1 | 0 | 0 | 0 | 0 | 7 | 87.50% |
| Total | 49 | 34 | 18 | 17 | 6 | 31 | 7 | 82.72% |

Table S3. 19 bioclimatic indicators of environmental indicators in this study.

| Name | Mean | Unit |
| --- | --- | --- |
| Bio_1 | Annual mean temperature | °C |
| Bio_2 | Mean diurnal range | °C |
| Bio_3 | Isothermality (BIO2/BIO7) (* 100) | % |
| Bio_4 | Temperature seasonality (standard deviation *100) | °C |
| Bio_5 | Max temperature of warmest month | °C |
| Bio_6 | Min temperature of coldest month | °C |
| Bio_7 | Temperature annual range (BIO5-BIO6) | °C |
| Bio_8 | Mean temperature of wettest quarter | °C |
| Bio_9 | Mean temperature of driest quarter | °C |
| Bio_10 | Mean temperature of warmest quarter | °C |
| Bio_11 | Mean temperature of coldest quarter | °C |
| Bio_12 | Annual precipitation | mm |
| Bio_13 | Precipitation of wettest month | mm |
| Bio_14 | Precipitation of driest month | mm |
| Bio_15 | Precipitation seasonality (coefficient of variation) | 1 |
| Bio_16 | Precipitation of wettest quarter | mm |
| Bio_17 | Precipitation of driest quarter | mm |
| Bio_18 | Precipitation of warmest quarter | mm |
| Bio_19 | Precipitation of coldest quarter | mm |


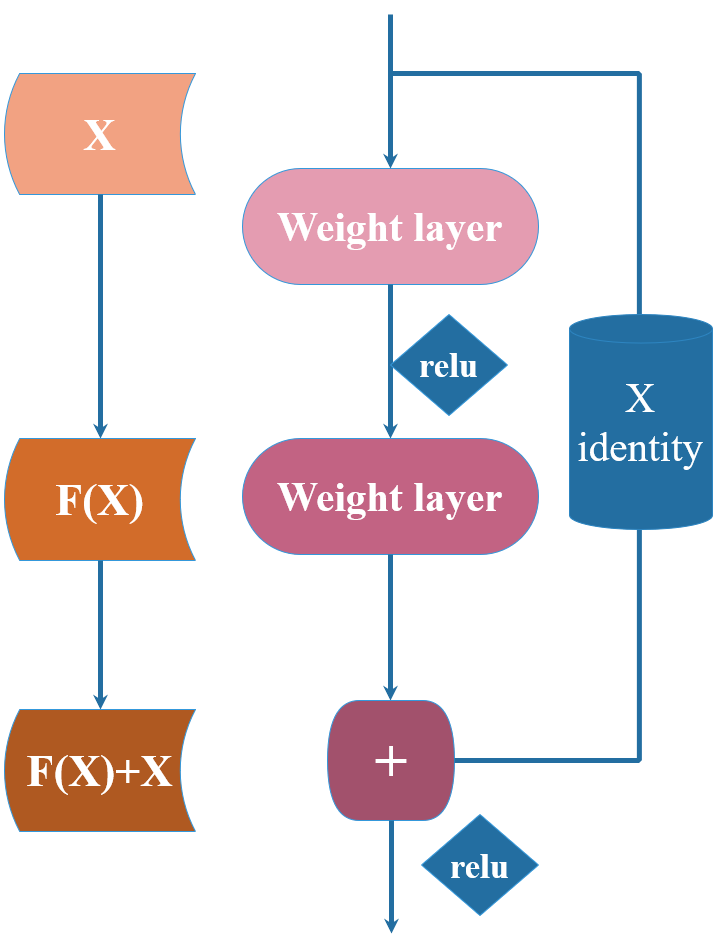


Figure S1


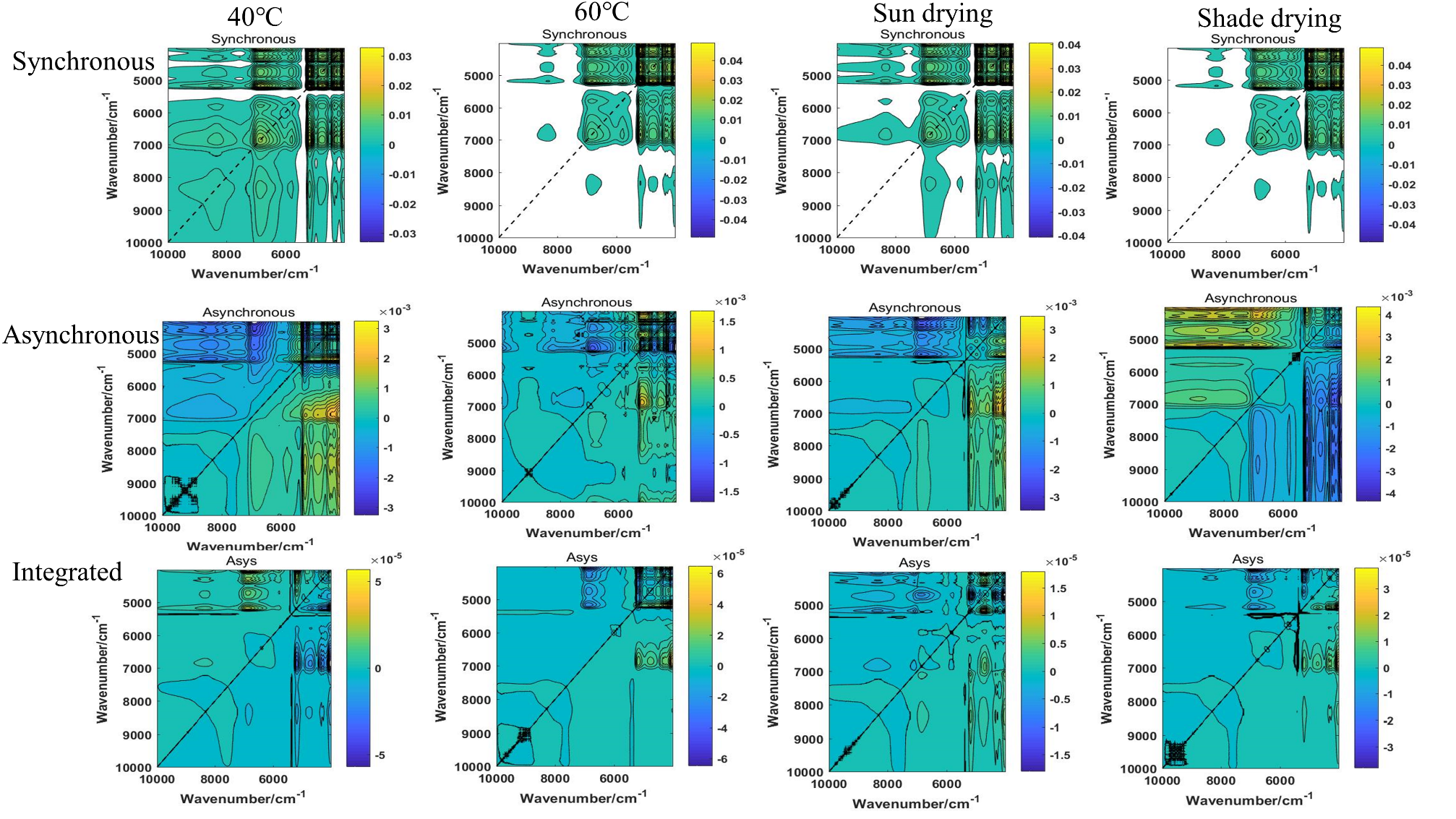


Figure S2


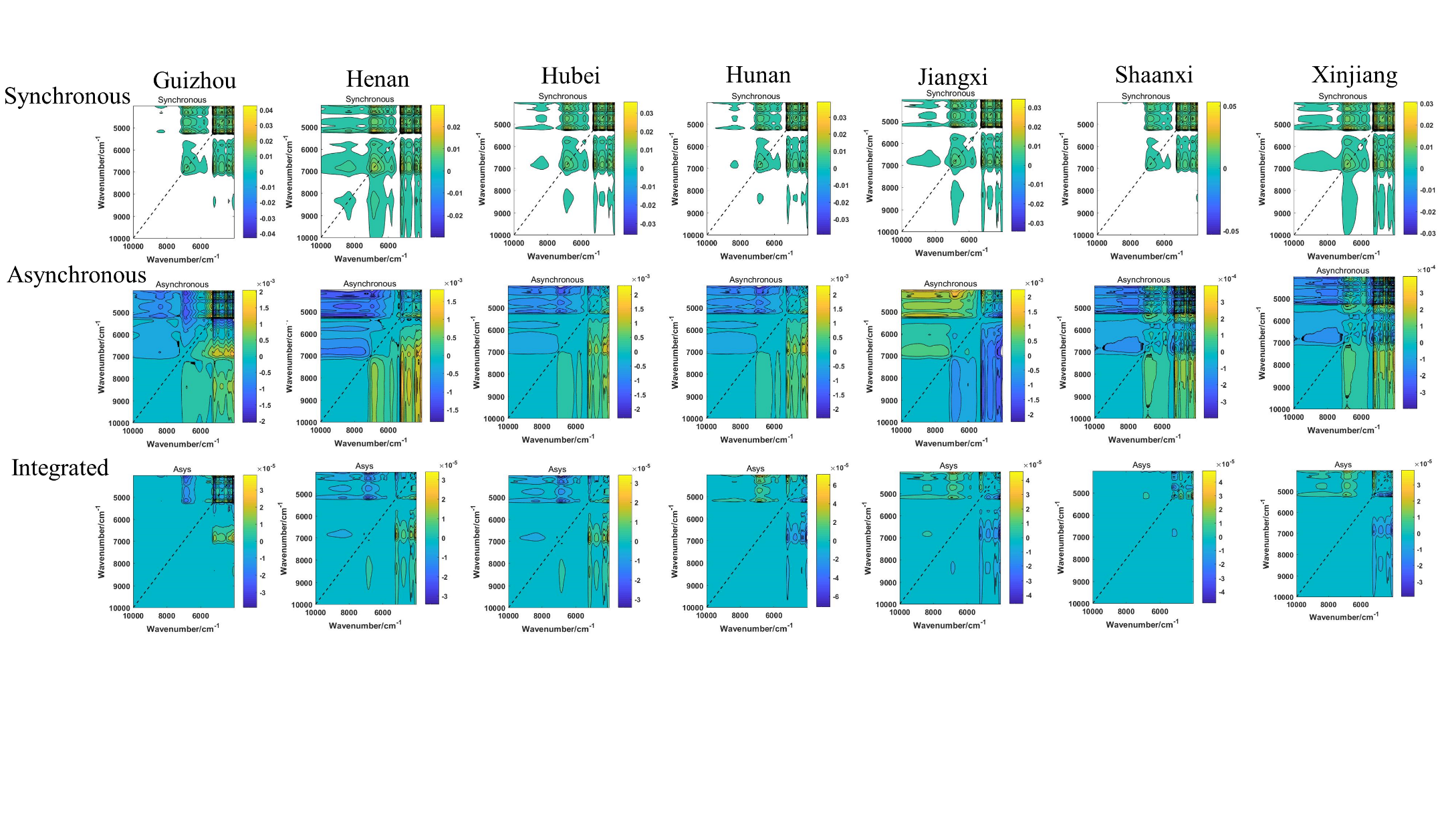


Figure S3


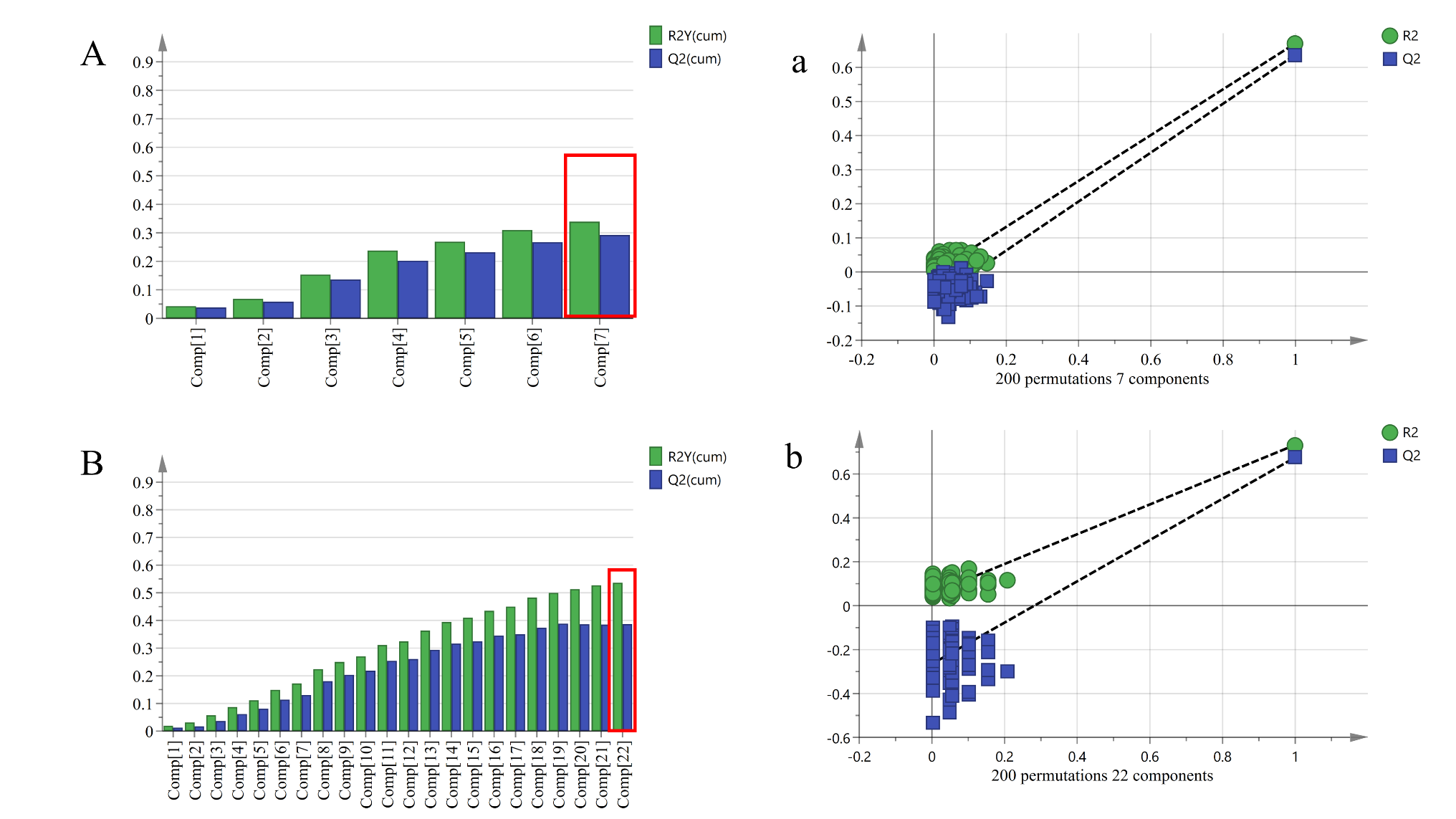


Figure S4


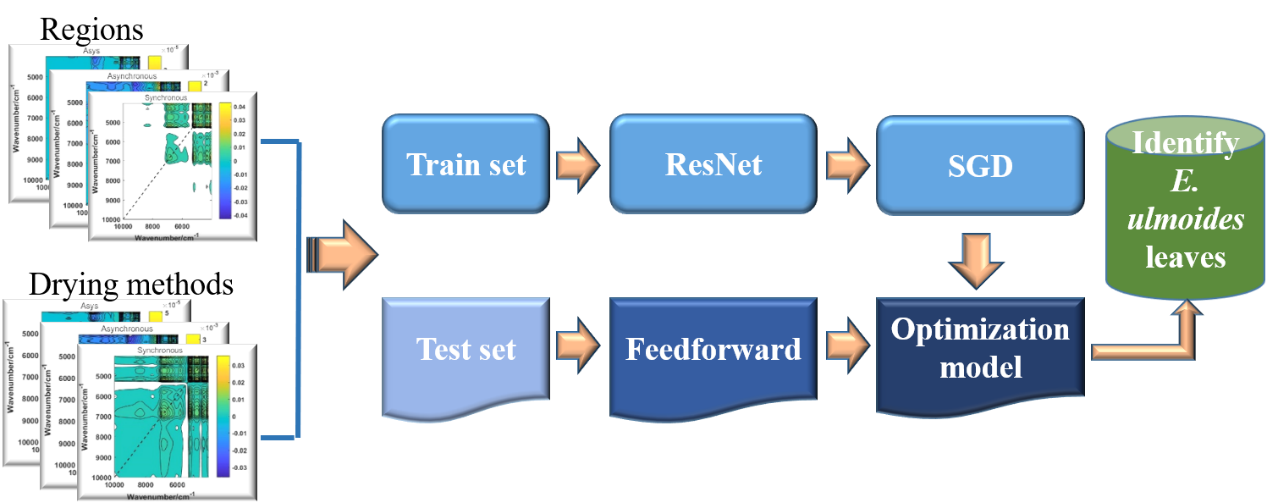


Figure S5


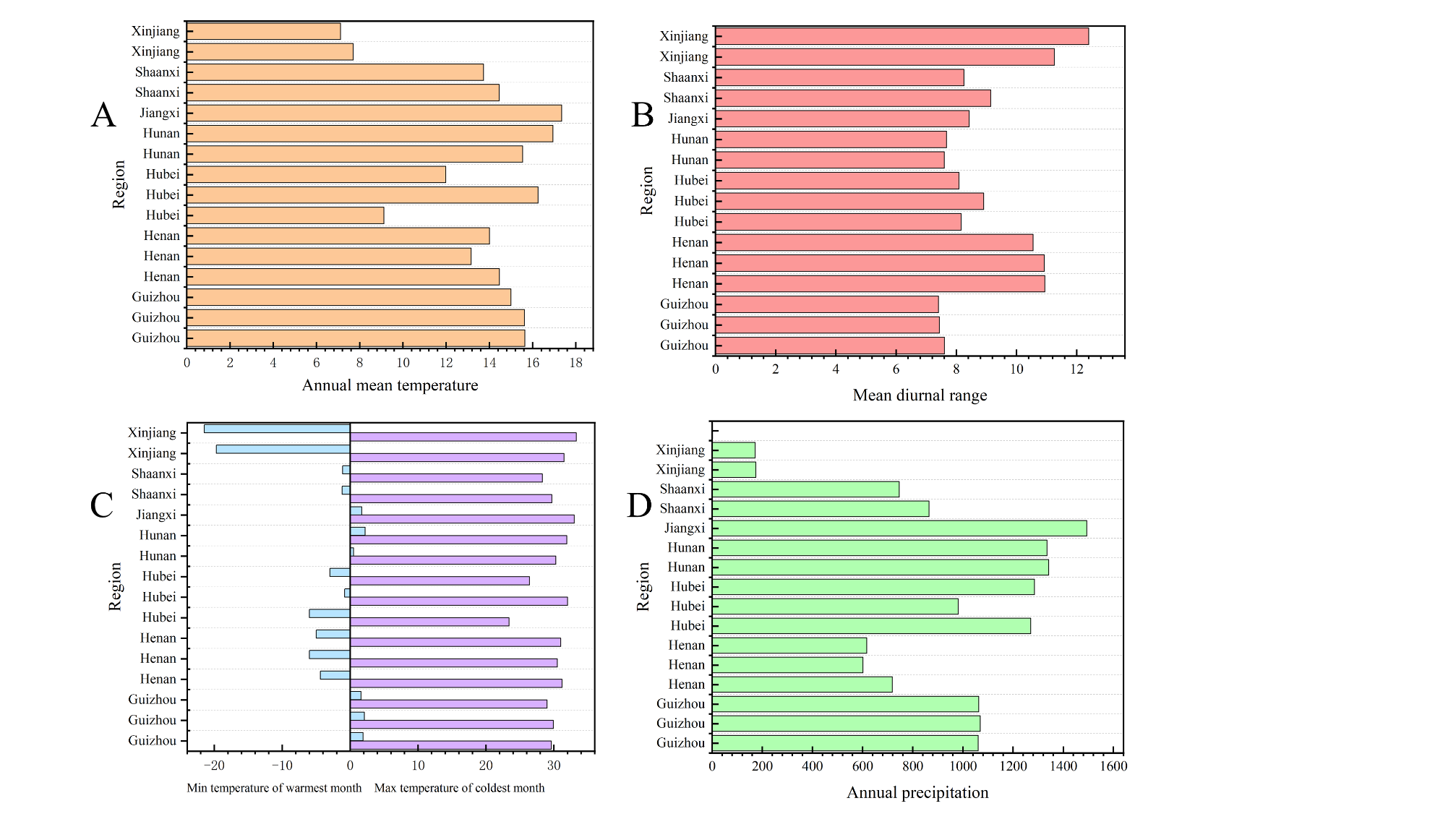


Figure S6


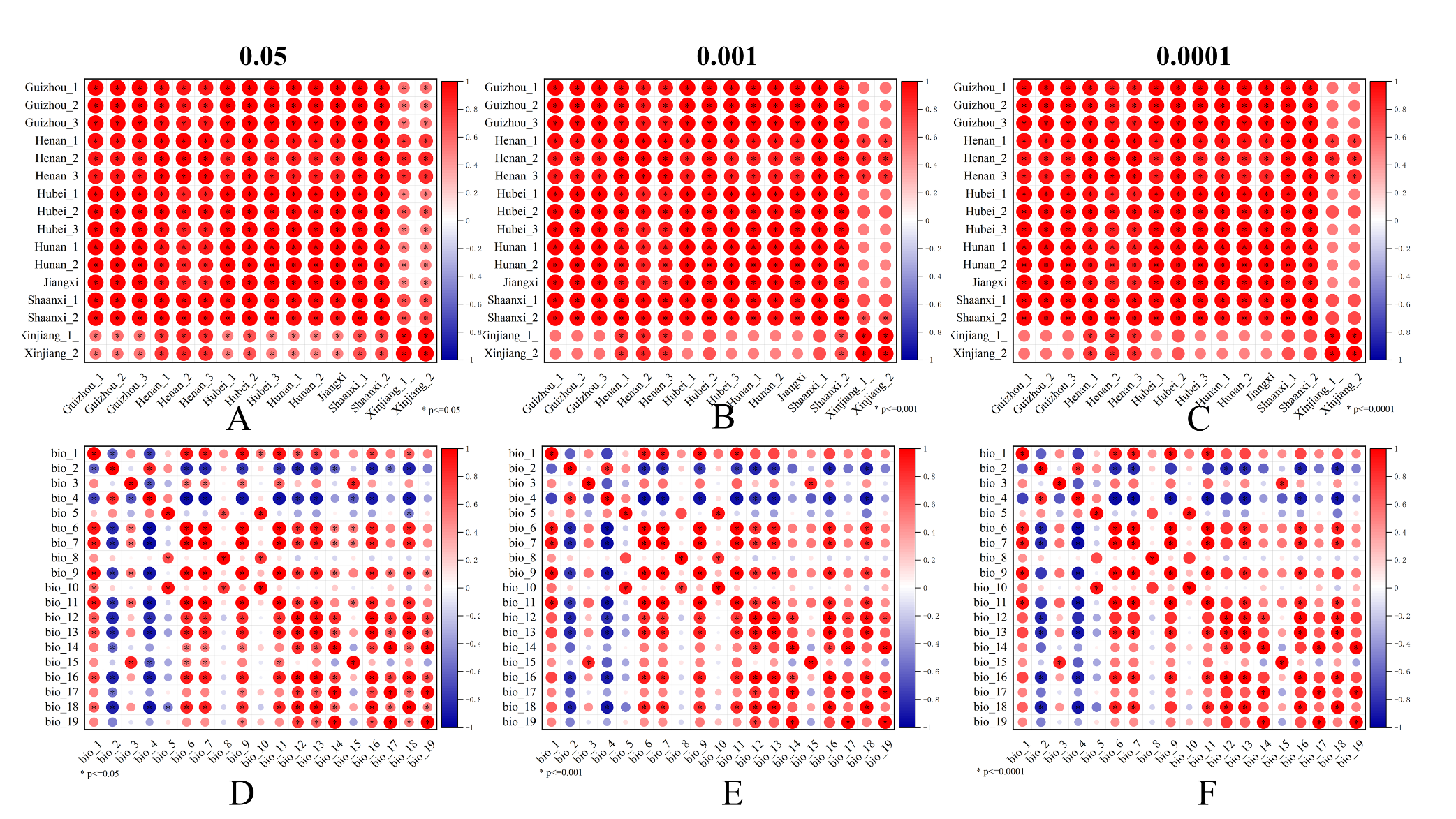


Figure S7
